# A robust poly-reference frequency-domain identification method to extract dynamic properties from vibration data

**DOI:** 10.1038/s44172-023-00122-y

**Published:** 2023-10-17

**Authors:** Sandro Diord Rescinho Amador, Rune Brincker

**Affiliations:** 1 Technical Univeristy of Denmark, Kgs. Lyngby, Denmark; 2 Brincker Monitoring ApS, Copenhagen, Denmark

**Keywords:** Aerospace engineering, Electrical and electronic engineering, Civil engineering, Mechanical engineering, Energy infrastructure

## Abstract

When performing vibration tests on structural systems, engineers face the challenge of extracting the dynamic properties from the measured data in an accurate and robust manner. Though several methods exist for this purpose, in some circumstances, they fail to provide clear estimates for these properties, particularly when applied to noise-contaminated data. Here we propose a robust and accurate method formulated in frequency-domain modal model for extracting dynamic properties from vibration data. The method is applied to three application examples, namely the vibrations simulated with the aid of a finite element model and the real-life vibration measurements of a platform specimen and of a full-scale concrete heritage court building. Its performance is thereafter assessed by the so-called stabilization diagrams, the relative error between estimated and exact properties, the modal assurance criterion, and by comparing the synthesized frequency functions to their measured counterparts. This assessment shows that the proposed approach tends to provide clearer and more accurate identification results than those from the state-of-the-art identification methods.

## Abstract (web summary)

Poly-reference system identification algorithms are mathematical tools to accurately estimate the dynamic properties of structural systems from vibration measurements. However, in some circumstance, they have a high computational cost and uncertainty. Amador and Brincker report an accurate and robust poly-reference formulation that can be easily implemented and demonstrate its performance by means of simulated and real-world application examples, including using a 15-storey heritage building.

## Introduction

Structural dynamics is a fundamental subject area that played a very relevant role in the industrial modernization process occurred in the last decades. This is due to the fact that both theoretical and experimental structural dynamics cut across different areas such as civil, mechanical, electrical and aerospace engineering. Thanks to the advancements in experimental structural dynamics over the last 50 years, cars, buildings, long span bridges, airplanes, rockets, electric circuits, etc., are constructed safer and with much better dynamic performance nowadays than ever before. In this context, Experimental and Operational Modal Analysis (EMA and OMA) are key analysis tools in determining accurately the dynamic properties of structural systems by means of vibration tests. The main goal of both EMA and OMA is to extract the physics, i.e., the dynamic (or modal) properties of the tested structural systems from the measurements acquired in vibration tests. While in classic EMA, both the vibration responses and the excitation forces are needed to extract this information, in OMA technology, only the measured vibration responses are required to estimate such properties.

The former is better suited to the analysis of vibration measurements collected under laboratory conditions, i.e., to mechanical, electrical and aerospace structural systems, whereas the latter is more appropriate to the analysis of large outdoor structures such as civil engineering structures. When designing structural systems that are inherently exposed to vibrations due to their operating and/or surrounding environmental conditions, engineers are challenged to accurately determine their fundamental dynamic properties by means of either EMA or OMA. This information is crucial, among other purposes, to subsequentely assess their structural performance, comfort and safety under operating conditions, as well as to improve and optimize their structural design. Over the last five decades, there have been groundbreaking advancements in modern experimental structural dynamics, namely in EMA and OMA.

In the late seventies, the Ibrahim Time Domain (ITD)^1,2^ was introduced to the modal analysis community as the first single-reference Least Squares (LS) modal identification technique capable of handling multiple output measurements at once. Later on, the poly-reference LS Complex Exponential^3,4^ was invented. Such an algorithm consists of a poly-reference LS modal identification technique, meaning that it is capable of taking into account multiple input and multiple output measurements at once in the identification process. Shortly after the invention of the poly-reference LS Complex Exponential, the ITD was reformulated into a poly-reference LS technique^5^. Though the ITD and the poly-reference LS Complex Exponential were the first poly-reference modal analysis algorithms ever invented, to this very day, they are still regarded by many as some of the most accurate and robust modal analysis algorithms available.

The invention of such time-domain poly-reference algorithms unleashed a revolution in experimental dynamics that would continue with the subsequent development of the so-called subspace-based methods such as the Eigensystem Realization Algorithm^6–8^ and the Stochastic Subspace Identification technique^9,10^. Recently, around the mid-2000s, this revolution would be consolidated with the formulation of the frequency-domain poly-reference technique known as poly-reference Least Squares Complex Frequency-domain (pLSCF)^11,12^. The pLSCF is nowadays deemed as a standard in EMA and OMA due to its ability to provide very accurate estimates for the modal properties and create clear stabilization diagrams that allow the physical modal properties to be easily sorted out of the numerical ones. Because the computation of the pLSCF normal matrices is time- and resource-consuming, a fast implementation of the algorithm can be carried out using a strategy similar to that described in ref. ^13^.

At around the same time as the invention of the pLSCF technique, other achievements in non-parametric modal parameter estimation led to the consolidation of OMA as a reliable modal analysis tool to estimate the dynamic properties of structural systems from output-only vibration measurements. The most relevant of these achievements were the inventions of the Complex Modal Indication Function^14,15^ and the Frequency Domain Decomposition Technique^16–18^, and the realization that all the parametric poly-reference LS techniques initially formulated for EMA can be perfectly extended to output-only modal parameter estimation, i.e., to OMA^19^. This realization allowed engineers to perform vibration tests in very large structural systems in a much easier, cheaper, faster and more convenient manner^20^. Moreover, the invention of OMA led to another breakthrough in structural dynamics, which is the possibility of automatically tracking the damage evolution in such structural systems over their service lifetime.

The so-called vibration-based structural health monitoring, among other benefits, allows minimizing the risks of structural and serviceability failures, as well as reducing the maintenance costs^21,22^ by averting, for instance, unnecessary structural inspections. The underlying idea of vibration-based structural health monitoring is that, since the dynamic properties are intrinsically related to the strength or stiffness of the monitored structural systems, any change due to material degradation fatigue, etc., entails a change in the monitored dynamic properties. In this context, accurate and robust modal parameter estimation techniques play a crucial role in extracting the dynamic properties from the vibration data continuously collected in a structural health monitoring campaign. More recently, important achievements in non-linear modal identification algorithms, namely with the invention of the non-linear LS identification techniques such as the poly-reference Maximum Likelihood Based algorithms^23–25^, have occurred. These techniques have been increasingly used in classic EMA to improve the precision of estimates provided by the LS-based techniques when they are not accurate enough. The idea is to use the estimates from, for instance, a poly-reference LS-based algorithm as starting guess to iteratively improve their accuracy^21,26^.

In this paper, a poly-reference parametric modal identification technique is proposed. The approach consists of a poly-reference Complex Frequency-domain method formulated in the Modal Model, hence the acronym pCF, and is formulated in the frequency domain based on the ITD principles. The basic idea behind the ITD technique is to extract the dynamics properties by comparing the time-domain free decay function evaluated at two adjacent discrete time steps. The proposed pCF, on the other hand, is derived by following a similar strategy, but using frequency-domain functions as primary data. It is worth highlighting that, despite the similarity in terms of basic principle, the invented pCF is completely original in that (1) it is formulated in the frequency domain, and (2) it is derived based on the Z-Transform of the time-domain free decay function with a discrete-time shift. The advantages of the pCF with regard to other existing frequency-domain techniques are surprisingly outstanding. These advantages include, for instance, increased robustness in sorting the physical modal properties from the numerical ones and easy practical implementation.

In addition to the aforementioned benefits, the derivation of the system matrices of the pCF technique opens the doors for the development of other subspace-driven modal identification techniques formulated in frequency-domain state-space model, as briefly described in ref. ^27^. In order to illustrate its benefits from a practical perspective, the performance of the pCF technique is herein assessed by means of three application examples, e.g., a simulated input-output and two real-life output-only vibration tests.

## Methods

In experimental modal analysis, the main goal is to extract the modal properties from the vibration measurements collected in vibration tests of structural systems. The modal properties extraction (or modal identification) can be carried out either in the time domain or frequency domain. In the former, a free decay function is normally used as primary data. In this case, the modal properties are computed basically by fitting an analytical model to the free decay measurements. The impulse response function of a structural system with general viscous damping in continuous time containing the information of *N*_*i*_ inputs and *N*_*o*_ outputs can be modeled by the so-called time-domain modal model^28^ given by

1 $${{{{{{{\bf{Y}}}}}}}}\left(t\right)=\mathop{\sum }\limits_{i=1}^{{n}_{c}}{{{{{{{{\boldsymbol{\phi }}}}}}}}}_{i}{{{{{{{{\boldsymbol{\gamma }}}}}}}}}_{i}^{T}{e}^{{\lambda }_{i}t}+{{{{{{{{\boldsymbol{\phi }}}}}}}}}_{i}^{* }{{{{{{{{\boldsymbol{\gamma }}}}}}}}}_{i}^{H}{e}^{{\lambda }_{i}^{* }t}$$

where (•)^*T*^ and (•)^*H*^ denote the transpose and the complex conjugate transpose (Hermitian), respectively, and (•)^*^ the complex conjugate; $t\in {\mathbb{R}}$ is the continuous time; *n*_*c*_ is the number of physical vibration modes, which corresponds to a model order (i.e., the number of physical modes plus their complex conjugates) of *n* = 2*n*_*c*_; ${{{{{{{{\boldsymbol{\phi }}}}}}}}}_{r}\in {{\mathbb{C}}}^{{N}_{o}\times 1}$ and ${{{{{{{{\boldsymbol{\gamma }}}}}}}}}_{r}\in {{\mathbb{C}}}^{{N}_{i}\times 1}$ are, respectively, the mode shape and the modal participation factor vectors, and ${\lambda }_{r}\in {\mathbb{C}}$ the continuous-time poles, which are related to the natural frequencies in rads/s, *ω*_*i*_, and damping ratios, *ξ*_*i*_, as

2 $${\lambda }_{i},{\lambda }_{i}^{* }=-{\xi }_{i}{\omega }_{i}\pm j\sqrt{1-{\xi }_{i}^{2}}{\omega }_{i}$$

with $j=\sqrt{-1}$ designating the imaginary unit, and *ω*_*i*_ = 2*π* *f*_*i*_ the circular natural frequency in rad/s which can be expressed as function of the natural frequency in Hertz (cycles/s) *f*_*i*_. The impulse response matrix in continuous time, as in Eq. (1), can be reformulated into a more general form to model either a time delay or advance. If the latter is considered, Eq. (1) can be re-written as

3 $${{{{{{{\bf{Y}}}}}}}}(t+\tau )=\mathop{\sum }\limits_{i=1}^{{n}_{c}}{{{{{{{{\boldsymbol{\phi }}}}}}}}}_{i}{{{{{{{{\boldsymbol{\gamma }}}}}}}}}_{i}^{T}{e}^{{\lambda }_{i}(t+\tau )}+{{{{{{{{\boldsymbol{\phi }}}}}}}}}_{i}^{* }{{{{{{{{\boldsymbol{\gamma }}}}}}}}}_{i}^{H}{e}^{{\lambda }_{i}^{* }(t+\tau )}$$

where *τ* is a forward time shift in continuous time introduced in the classic impulse response matrix to model a time advance with respect to *t*. In practice, vibration tests are conducted in discrete time, meaning that the vibration data is recorded with a sampling interval, Δ*t*. Therefore, if the modal properties are to be extracted from the sampled vibration data, Eq. (3) needs to be re-written in discrete time, yielding

4 $${{{{{{{{\bf{Y}}}}}}}}}_{k+r}=\mathop{\sum }\limits_{i=1}^{{n}_{c}}{{{{{{{{\boldsymbol{\phi }}}}}}}}}_{i}{{{{{{{{\boldsymbol{\gamma }}}}}}}}}_{i}^{T}{e}^{{\lambda }_{i}(k+r){{\Delta }}t}+{{{{{{{{\boldsymbol{\phi }}}}}}}}}_{i}^{* }{{{{{{{{\boldsymbol{\gamma }}}}}}}}}_{i}^{H}{e}^{{\lambda }_{i}^{* }(k+r){{\Delta }}t}$$

where the index *k* denotes a discrete time instant *t* = *k*Δ*t* at which a continuous time signal is sampled and *τ* = *r*Δ*t* a discrete forward time shift. Eq. (4) can be written in a compact matrix equation, as

5 $${{{{{{{{\bf{Y}}}}}}}}}_{k+r}={{{{{{{\boldsymbol{\Phi }}}}}}}}{{{{{{{{\boldsymbol{\Lambda }}}}}}}}}^{k+r}{{{{{{{{\boldsymbol{\Gamma }}}}}}}}}^{T}$$

where ${{{{{{{\boldsymbol{\Phi }}}}}}}}\in {{\mathbb{C}}}^{{N}_{o}\times {n}}$ is the mode shape matrix, ${{{{{{{\boldsymbol{\Lambda }}}}}}}}\in {{\mathbb{C}}}^{{n}\times {n}}$ is a diagonal matrix containing the discreet-time poles, ${\mu }_{r}={e}^{{\lambda }_{r}{{\Delta }}t}$ and ${{{{{{{\boldsymbol{\Gamma }}}}}}}}\in {{\mathbb{C}}}^{{N}_{i}\times {n}}$ is the modal participation matrix. From Eq. (4), it is straightforward to prove that such matrices are given by

6 $${{{{{{{\boldsymbol{\Phi }}}}}}}} 	=\left[\begin{array}{ccccc}{{{{{{{{\boldsymbol{\phi }}}}}}}}}_{1}&{{{{{{{{\boldsymbol{\phi }}}}}}}}}_{1}^{* }&\cdots \,&{{{{{{{{\boldsymbol{\phi }}}}}}}}}_{{n}_{c}}&{{{{{{{{\boldsymbol{\phi }}}}}}}}}_{{n}_{c}}^{* }\end{array}\right]\in {{\mathbb{C}}}^{{N}_{o}\times {n}}\\ {{{{{{{\boldsymbol{\Gamma }}}}}}}} 	=\left[\begin{array}{ccccc}{{{{{{{{\boldsymbol{\gamma }}}}}}}}}_{1}&{{{{{{{{\boldsymbol{\gamma }}}}}}}}}_{1}^{* }&\cdots \,&{{{{{{{{\boldsymbol{\gamma }}}}}}}}}_{{n}_{c}}&{{{{{{{{\boldsymbol{\gamma }}}}}}}}}_{{n}_{c}}^{* }\end{array}\right]\in {{\mathbb{C}}}^{{N}_{i}\times {n}}\\ {{{{{{{\boldsymbol{\Lambda }}}}}}}} 	={{{{{{{\rm{diag}}}}}}}}\left(\left[\begin{array}{ccccc}{\mu }_{1}&{\mu }_{1}^{* }&\cdots \,&{\mu }_{{n}_{c}}&{\mu }_{{n}_{c}}^{* }\end{array}\right]\right)\in {{\mathbb{C}}}^{{n}\times {n}}$$

where ${{{{{{{\rm{diag}}}}}}}}\left(\bullet \right)$ denotes the diagonal matrix operator. The impulse response model with a forward time shift, as in Eq. (5), can be converted from the time to the frequency domain by making use of the Laplace Transform, also known as S-Transform. Taking the Laplace Transform of Eq. (5), yields

7 $${z}^{r}{{{{{{{\bf{H}}}}}}}}(s)={{{{{{{\boldsymbol{\Phi }}}}}}}}{{{{{{{{\boldsymbol{\Lambda }}}}}}}}}^{r}{\left[{{{{{{{\bf{I}}}}}}}}s-{{{{{{{{\boldsymbol{\Lambda }}}}}}}}}_{c}\right]}^{-1}{{{{{{{{\boldsymbol{\Gamma }}}}}}}}}^{T}$$

which is so-called transfer function matrix with *s* = *j* *ω* designating the Laplace domain variable, *z* = *e* ^*s*Δ*t*^ = *e* ^*jω*Δ*t*^ the Z-domain variable, ${{{{{{{\bf{I}}}}}}}}\in {{\mathbb{R}}}^{{n}\times {n}}$ the identity matrix and

8 $${{{{{{{{\boldsymbol{\Lambda }}}}}}}}}_{c}={{{{{{{\rm{diag}}}}}}}}\left(\left[\begin{array}{ccccc}{\lambda }_{1}&{\lambda }_{1}^{* }&\cdots \,&{\lambda }_{{n}_{c}}&{\lambda }_{{n}_{c}}^{* }\end{array}\right]\right)\in {{\mathbb{C}}}^{{n}\times {n}}$$

a diagonal matrix containing the continuous time poles, ${\lambda }_{i},{\lambda }_{i}^{* }$. Eq. (7) is better known in its partial fraction form

9 $${z}^{r}{{{{{{{\bf{H}}}}}}}}(s)=\mathop{\sum }\limits_{i=1}^{{n}_{c}}\frac{{{{{{{{\boldsymbol{{\phi }}}}}}}_{i}}}{{{{{{{{\boldsymbol{{\gamma }}}}}}}_{i}}}}^{T}{\mu }_{i}^{r}}{s-{\lambda }_{i}}+\frac{{{{{{{{{\boldsymbol{{\phi }}}}}}}_{i}}}}^{* }{{{{{{{{\boldsymbol{{\gamma }}}}}}}_{i}}}}^{H}{\left({\mu }_{i}^{* }\right)}^{r}}{s-{\lambda }_{i}^{* }}$$

which is the Laplace Transform of the impulse response function in partial form with a forward time shift as in Eq. (4). If one considers the particular case of a zero forward time shift (e.g., *r* = 0), Eq. (9) reduces to the so-called transfer function in Laplace domain given by

10 $${{{{{{{\bf{H}}}}}}}}(s)=\mathop{\sum }\limits_{i=1}^{{n}_{c}}\frac{{{{{{{{\boldsymbol{{\phi }}}}}}}_{i}}}{{{{{{{{\boldsymbol{{\gamma }}}}}}}_{i}}}}^{T}}{s-{\lambda }_{i}}+\frac{{{{{{{{{\boldsymbol{{\phi }}}}}}}_{i}}}}^{* }{{{{{{{{\boldsymbol{{\gamma }}}}}}}_{i}}}}^{H}}{s-{\lambda }_{i}^{* }}$$

The impulse response function with a forward time shift, as in Eq. (5), can also be converted to frequency domain by means of the Z-Transform. By following an approach similar to the S-Transform previously described, the following time-shifted transfer function can be derived

11 $${z}^{r}{{{{{{{\bf{H}}}}}}}}(z)={{{{{{{\boldsymbol{\Phi }}}}}}}}{{{{{{{{\boldsymbol{\Lambda }}}}}}}}}^{r}{\left[{{{{{{{\bf{I}}}}}}}}z-{{{{{{{\boldsymbol{\Lambda }}}}}}}}\right]}^{-1}{{{{{{{{\boldsymbol{\Gamma }}}}}}}}}^{T}$$

which corresponds to the following function in partial fraction form

12 $${z}^{r}{{{{{{{\bf{H}}}}}}}}(z)=\mathop{\sum }\limits_{i=1}^{{n}_{c}}\frac{{{{{{{{\boldsymbol{{\phi }}}}}}}_{i}}}{{{{{{{{\boldsymbol{{\gamma }}}}}}}_{i}}}}^{T}{\mu }_{i}^{r}}{z-{\mu }_{i}}+\frac{{{{{{{{{\boldsymbol{{\phi }}}}}}}_{i}}}}^{* }{{{{{{{{\boldsymbol{{\gamma }}}}}}}_{i}}}}^{H}{({\mu }_{i}^{* })}^{r}}{z-{\mu }_{i}^{* }}$$

Eqs. (11) and (12) are central in the formulation of the proposed pCF detailed described in the next section. These equations follow from the property of the Z-transform in which a forward time shift of *r* in the time domain corresponds to a multiplication by *z* ^*r*^ in the frequency domain, i.e.,

$${\mathbf{Y}}_{k+r}={\mathbf{\Phi}}{\mathbf{\Lambda}}^{k+r}{\mathbf{\Gamma}}^{T} \mathop{\Leftrightarrow}\limits^{{ {{\mathbf{Z}}}-{\mathbf{Transf}}}} \, z^r{{\mathbf{H}}}(z)={\mathbf{\Phi}}{\mathbf{\Lambda}}^r \left[{\mathbf{I}}z-{\mathbf{\Lambda}}\right]^{-1}{\mathbf{\Gamma}}^T$$

### The poly-reference Complex Frequency (pCF) technique (a proposed approach)

Despite the fact that derivation of the pCF can be carried out both with the S- and Z-Transform, the derivation with the latter is more obvious and straightforward, and leads to a unique solution. The derivation of the pCF technique with Z-Transform starts from the Z-domain transfer function with a general time shift as in Eq. (11). Writing down such equation for a set of *n* forward time shifts, i.e., for *r* = 0, …, *n*, the following set of equations is  obtained

13 $$\begin{array}{l}{{{{{{{\bf{H}}}}}}}}(z)={{{{{{{\boldsymbol{\Phi }}}}}}}}{\left({{{{{{{\bf{I}}}}}}}}z-{{{{{{{\boldsymbol{\Lambda }}}}}}}}\right)}^{-1}{{{{{{{{\boldsymbol{\Gamma }}}}}}}}}^{T}\\ z{{{{{{{\bf{H}}}}}}}}(z)={{{{{{{\boldsymbol{\Phi }}}}}}}}{{{{{{{\boldsymbol{\Lambda }}}}}}}}{\left({{{{{{{\bf{I}}}}}}}}z-{{{{{{{\boldsymbol{\Lambda }}}}}}}}\right)}^{-1}{{{{{{{{\boldsymbol{\Gamma }}}}}}}}}^{T}\\ \vdots \\ {z}^{n}{{{{{{{\bf{H}}}}}}}}(z)={{{{{{{\boldsymbol{\Phi }}}}}}}}{{{{{{{{\boldsymbol{\Lambda }}}}}}}}}^{n}{\left({{{{{{{\bf{I}}}}}}}}z-{{{{{{{\boldsymbol{\Lambda }}}}}}}}\right)}^{-1}{{{{{{{{\boldsymbol{\Gamma }}}}}}}}}^{T}\end{array}$$

Now, combining the obtained equations into a single matrix expression, yields

14 $$\left[\begin{array}{c}{{{{{{{\bf{H}}}}}}}}(z)\\ z{{{{{{{\bf{H}}}}}}}}(z)\\ \vdots \\ {z}^{n}{{{{{{{\bf{H}}}}}}}}(z)\\ \end{array}\right]=\left[\begin{array}{c}{{{{{{{\boldsymbol{\Phi }}}}}}}}\\ {{{{{{{\boldsymbol{\Phi }}}}}}}}{{{{{{{\boldsymbol{\Lambda }}}}}}}}\\ \vdots \\ {{{{{{{\boldsymbol{\Phi }}}}}}}}{{{{{{{{\boldsymbol{\Lambda }}}}}}}}}^{n}\\ \end{array}\right]{\left[{{{{{{{\bf{I}}}}}}}}z-{{{{{{{\boldsymbol{\Lambda }}}}}}}}\right]}^{-1}{{{{{{{{\boldsymbol{\Gamma }}}}}}}}}^{T}$$

or simply

15 $$\left[\begin{array}{c}{{{{{{{\bf{H}}}}}}}}(z)\\ z{{{{{{{\bf{H}}}}}}}}(z)\\ \vdots \\ {z}^{n}{{{{{{{\bf{H}}}}}}}}(z)\\ \end{array}\right]={{{{{{{\boldsymbol{\Psi }}}}}}}}{\left[{{{{{{{\bf{I}}}}}}}}z-{{{{{{{\boldsymbol{\Lambda }}}}}}}}\right]}^{-1}{{{{{{{{\boldsymbol{\Gamma }}}}}}}}}^{T}$$

with

16 $${{{{{{{\boldsymbol{\Psi }}}}}}}}=\left[\begin{array}{c}{{{{{{{\boldsymbol{\Phi }}}}}}}}\\ {{{{{{{\boldsymbol{\Phi }}}}}}}}{{{{{{{\boldsymbol{\Lambda }}}}}}}}\\ \vdots \\ {{{{{{{\boldsymbol{\Phi }}}}}}}}{{{{{{{{\boldsymbol{\Lambda }}}}}}}}}^{n}\\ \end{array}\right]\in {{\mathbb{R}}}^{{N}_{o}(n+1)\times {N}_{o}(n+1)}$$

At this point, it is worth highlighting that due to the merger of Eqs. (13) into (14), the dimensions of **Φ** and **Λ** become, respectively, *N*_*o*_ × *N*_*o*_(*n* + 1) and *N*_*o*_(*n* + 1) × *N*_*o*_(*n* + 1), which allows to estimate (*n* + 1)*N*_*o*_ vibration modes. Eq. (15) can be re-written as

17 $${{{{{{{{\boldsymbol{\Psi }}}}}}}}}^{-1}\left[\begin{array}{c}z{{{{{{{\bf{H}}}}}}}}(z)\\ {z}^{2}{{{{{{{\bf{H}}}}}}}}(z)\\ \vdots \\ {z}^{n+1}{{{{{{{\bf{H}}}}}}}}(z)\\ \end{array}\right]-{{{{{{{\boldsymbol{\Lambda }}}}}}}}{{{{{{{{\boldsymbol{\Psi }}}}}}}}}^{-1}\left[\begin{array}{c}{{{{{{{\bf{H}}}}}}}}(z)\\ z{{{{{{{\bf{H}}}}}}}}(z)\\ \vdots \\ {z}^{n}{{{{{{{\bf{H}}}}}}}}(z)\\ \end{array}\right]={{{{{{{{\boldsymbol{\Gamma }}}}}}}}}^{T}$$

Following a strategy similar to that used in the formulation of the ITD technique^1,2^, one can evaluate Eq. (17) for any neighboring frequency lines *ω*_*a*_ and *ω*_*b*_ (∀*ω*_*b*_ > *ω*_*a*_) separated by a single discrete frequency step Δ*ω*, giving

18 $${\boldsymbol{\Psi}}^{-1}\left[\begin{array}{c}{z}_{a}{\bf{H}}({z}_{a})\\ {z}_{a}^{2}{\bf{H}}({z}_{a})\\ \vdots \\ {z}_{a}^{n+1}{\bf{H}}({z}_{a})\\ \end{array}\right]-{\boldsymbol{\Lambda }}{\boldsymbol{\Psi }}^{-1}\left[\begin{array}{c}{\bf{H}}({z}_{a})\\ {z}_{a}{\bf{H}}({z}_{a})\\ \vdots \\ {z}_{a}^{n}{\bf{H}}({z}_{a})\\ \end{array}\right]={\boldsymbol{\Gamma }}^{T}$$

and

19 $${\boldsymbol{\Psi}}^{-1}\left[\begin{array}{c}{z}_{b}{\bf{H}}({z}_{b})\\ {z}_{b}^{2}{\bf{H}}({z}_{b})\\ \vdots \\ {z}_{b}^{n+1}{\bf{H}}({z}_{b})\\ \end{array}\right]-{\boldsymbol{\Lambda }}{\boldsymbol{\Psi }}^{-1}\left[\begin{array}{c}{\bf{H}}({z}_{b})\\ {z}_{b}{\bf{H}}({z}_{b})\\ \vdots \\ {z}_{b}^{n}{\bf{H}}({z}_{b})\\ \end{array}\right]={\boldsymbol{\Gamma }}^{T}$$

where ${z}_{a}={e}^{{s}_{a}{{\Delta }}t}={e}^{j{\omega }_{a}{{\Delta }}t}$ and ${z}_{b}={e}^{{s}_{b}{{\Delta }}t}={e}^{j{\omega }_{b}{{\Delta }}t}$ denote the Z-domain variable evaluated, respectively, at the frequency lines *ω*_*a*_ and *ω*_*b*_. Combining Eqs. (18) and (19), yields

20 $${{{{{{{{\boldsymbol{\Psi }}}}}}}}}^{-1}\left[\begin{array}{c}{z}_{b}{{{{{{{\bf{H}}}}}}}}({z}_{b})-{z}_{a}{{{{{{{\bf{H}}}}}}}}({z}_{a})\\ {z}_{b}^{2}{{{{{{{\bf{H}}}}}}}}({z}_{b})-{z}_{a}^{2}{{{{{{{\bf{H}}}}}}}}({z}_{a})\\ \vdots \\ {z}_{b}^{n+1}{{{{{{{\bf{H}}}}}}}}({z}_{b})-{z}_{a}^{n+1}{{{{{{{\bf{H}}}}}}}}({z}_{a})\\ \end{array}\right]={{{{{{{\boldsymbol{\Lambda }}}}}}}}{{{{{{{{\boldsymbol{\Psi }}}}}}}}}^{-1}\left[\begin{array}{c}{{{{{{{\bf{H}}}}}}}}({z}_{b})-{{{{{{{\bf{H}}}}}}}}({z}_{a})\\ {z}_{b}{{{{{{{\bf{H}}}}}}}}({z}_{b})-{z}_{a}{{{{{{{\bf{H}}}}}}}}({z}_{a})\\ \vdots \\ {z}_{b}^{n}{{{{{{{\bf{H}}}}}}}}({z}_{b})-{z}_{a}^{n}{{{{{{{\bf{H}}}}}}}}({z}_{a})\\ \end{array}\right]$$

From this point onwards, it is straightforward to formulate an eigenvalue problem based on Eq. (20). This is achieved, first, by writing down such equation for all the *N*_*f*_ discrete frequency lines in the frequency range of DC (*ω*_0_) to the Nyquist frequency (${\omega }_{{N}_{f}}$), i.e., for *ω*_*a*_ and *ω*_*b*_ ranging, respectively, from *ω*_0_ to ${\omega }_{{N}_{f}-1}$ and from *ω*_1_ to ${\omega }_{{N}_{f}}$. Then, combining the obtained equations corresponding to each pair of evaluated frequency values in a single matrix equation gives

21 $${{{{{{{{\boldsymbol{\Psi }}}}}}}}}^{-1}{{{{{{{{\bf{B}}}}}}}}}_{1}={{{{{{{\boldsymbol{\Lambda }}}}}}}}{{{{{{{{\boldsymbol{\Psi }}}}}}}}}^{-1}{{{{{{{{\bf{B}}}}}}}}}_{0}$$

with

22 $${{{{{{{{\bf{B}}}}}}}}}_{0}= \left[\begin{array}{cccc}{{{{{{{\bf{H}}}}}}}}({z}_{1})-{{{{{{{\bf{H}}}}}}}}({z}_{0})&{{{{{{{\bf{H}}}}}}}}({z}_{2})-{{{{{{{\bf{H}}}}}}}}({z}_{1})&\cdots \,&{{{{{{{\bf{H}}}}}}}}({z}_{{N}_{f}})-{{{{{{{\bf{H}}}}}}}}({z}_{{N}_{f}-1})\\ {z}_{1}{{{{{{{\bf{H}}}}}}}}({z}_{1})-{z}_{0}{{{{{{{\bf{H}}}}}}}}({z}_{0})&{z}_{2}{{{{{{{\bf{H}}}}}}}}({z}_{2})-{z}_{1}{{{{{{{\bf{H}}}}}}}}({z}_{1})&\cdots \,&{z}_{{N}_{f}}{{{{{{{\bf{H}}}}}}}}({z}_{{N}_{f}})-{z}_{{N}_{f}-1}{{{{{{{\bf{H}}}}}}}}({z}_{{N}_{f}-1})\\ \vdots &\vdots &\ddots &\vdots \\ {z}_{1}^{n}{{{{{{{\bf{H}}}}}}}}({z}_{1})-{z}_{0}^{n}{{{{{{{\bf{H}}}}}}}}({z}_{0})&{z}_{2}^{n}{{{{{{{\bf{H}}}}}}}}({z}_{2})-{z}_{1}^{n}{{{{{{{\bf{H}}}}}}}}({z}_{1})&\cdots \,&{z}_{{N}_{f}}^{n}{{{{{{{\bf{H}}}}}}}}({z}_{{N}_{f}})-{z}_{{N}_{f}-1}^{n}{{{{{{{\bf{H}}}}}}}}({z}_{{N}_{f}-1})\\ \end{array}\right] \in {{\mathbb{R}}}^{{N}_{o}(n+1)\times {N}_{i}({N}_{f}-1)}$$

and

23 $${{{{{{{{\bf{B}}}}}}}}}_{1}= \left[\begin{array}{cccc}{z}_{1}{{{{{{{\bf{H}}}}}}}}({z}_{1})-{z}_{0}{{{{{{{\bf{H}}}}}}}}({z}_{0})&{z}_{2}{{{{{{{\bf{H}}}}}}}}({z}_{2})-{z}_{1}{{{{{{{\bf{H}}}}}}}}({z}_{1})&\cdots \,&{z}_{{N}_{f}}{{{{{{{\bf{H}}}}}}}}({z}_{{N}_{f}})-{z}_{{N}_{f}-1}{{{{{{{\bf{H}}}}}}}}({z}_{{N}_{f}-1})\\ {z}_{1}^{2}{{{{{{{\bf{H}}}}}}}}({z}_{1})-{z}_{0}^{2}{{{{{{{\bf{H}}}}}}}}({z}_{0})&{z}_{2}^{2}{{{{{{{\bf{H}}}}}}}}({z}_{2})-{z}_{1}^{2}{{{{{{{\bf{H}}}}}}}}({z}_{1})&\cdots \,&{z}_{{N}_{f}}^{2}{{{{{{{\bf{H}}}}}}}}({z}_{{N}_{f}})-{z}_{{N}_{f}-1}^{2}{{{{{{{\bf{H}}}}}}}}({z}_{{N}_{f}-1})\\ \vdots &\vdots &\ddots &\vdots \\ {z}_{1}^{n+1}{{{{{{{\bf{H}}}}}}}}({z}_{1})-{z}_{0}^{n+1}{{{{{{{\bf{H}}}}}}}}({z}_{0})&{z}_{2}^{n+1}{{{{{{{\bf{H}}}}}}}}({z}_{2})-{z}_{1}^{n+1}{{{{{{{\bf{H}}}}}}}}({z}_{1})&\cdots \,&{z}_{{N}_{f}}^{n}{{{{{{{\bf{H}}}}}}}}({z}_{{N}_{f}})-{z}_{{N}_{f}-1}^{n}{{{{{{{\bf{H}}}}}}}}({z}_{{N}_{f}-1})\\ \end{array}\right] \in {{\mathbb{R}}}^{{N}_{o}(n+1)\times {N}_{i}({N}_{f}-1)}$$

designating the system matrices computed solely from the measured Frequency Response Function (FRF) and the Z-domain variable evaluated at all the frequency lines in the frequency range of DC to the Nyquist frequency ${\omega }_{{N}_{f}}$. It is worth noting that **B**_1_ is forward shifted in the time domain by Δ*t* with regard to **B**_0_ and that Eq. (21) can be solved for **Ψ** and **Λ** if it is post multiplied either by ${{{{{{{{\bf{B}}}}}}}}}_{0}^{H}$ or ${{{{{{{{\bf{B}}}}}}}}}_{1}^{H}$, giving

24 $${{{{{{{{\bf{B}}}}}}}}}_{1}{{{{{{{{\bf{B}}}}}}}}}_{0}^{H}{\left({{{{{{{{\bf{B}}}}}}}}}_{0}{{{{{{{{\bf{B}}}}}}}}}_{0}^{H}\right)}^{-1}={{{{{{{\boldsymbol{\Psi }}}}}}}}{{{{{{{\boldsymbol{\Lambda }}}}}}}}{{{{{{{{\boldsymbol{\Psi }}}}}}}}}^{-1}$$

or

25 $${{{{{{{{\bf{B}}}}}}}}}_{1}{{{{{{{{\bf{B}}}}}}}}}_{1}^{H}{\left({{{{{{{{\bf{B}}}}}}}}}_{0}{{{{{{{{\bf{B}}}}}}}}}_{1}^{H}\right)}^{-1}={{{{{{{\boldsymbol{\Psi }}}}}}}}{{{{{{{\boldsymbol{\Lambda }}}}}}}}{{{{{{{{\boldsymbol{\Psi }}}}}}}}}^{-1}$$

However, similarly to the ITD technique, rather than considering either of these solutions alone, the Double Least Squares approach^19,29^ is used to formulate the following eigenvalue problem

26 $${{{{{{{\bf{B}}}}}}}}={{{{{{{\boldsymbol{\Psi }}}}}}}}{{{{{{{\boldsymbol{\Lambda }}}}}}}}{{{{{{{{\boldsymbol{\Psi }}}}}}}}}^{-1}$$

with

27 $${{{{{{{\bf{B}}}}}}}}=\frac{1}{2}\left({{{{{{{{\bf{B}}}}}}}}}_{1}{{{{{{{{\bf{B}}}}}}}}}_{0}^{H}{\left({{{{{{{{\bf{B}}}}}}}}}_{0}{{{{{{{{\bf{B}}}}}}}}}_{0}^{H}\right)}^{-1}+{{{{{{{{\bf{B}}}}}}}}}_{1}{{{{{{{{\bf{B}}}}}}}}}_{1}^{H}{\left({{{{{{{{\bf{B}}}}}}}}}_{0}{{{{{{{{\bf{B}}}}}}}}}_{1}^{H}\right)}^{-1}\right)\in {{\mathbb{R}}}^{{N}_{o}(n+1)\times {N}_{o}(n+1)}$$

computed as a linear combination of Eqs. (24) and (25). Once the eigenvalue problem (26) is solved, the mode shape matrix, **Φ**, is determined as the first *N*_*o*_ rows of **Ψ** and the continuous time poles, *λ*_*i*_, are retrieved from the diagonal of **Λ**.

#### Implementation and stabilization

Because **B**_0_ and **B**_1_ in Eq. (27) are complex matrices, the estimation of the modal parameters according to Eq. (26) yields mode shape vectors and poles not occurring in complex conjugate pairs. If modal shape vectors and poles occurring in complex conjugate pairs are preferred, the modal properties should then be computed using the following expression

28 $${{{{{{{{\bf{B}}}}}}}}}_{{{{{{{{\rm{re}}}}}}}}}={{{{{{{\boldsymbol{\Psi }}}}}}}}{{{{{{{\boldsymbol{\Lambda }}}}}}}}{{{{{{{{\boldsymbol{\Psi }}}}}}}}}^{-1}\in {{\mathbb{R}}}^{{N}_{o}(n+1)\times {N}_{o}(n+1)}$$

where ${{{{{{{{\bf{B}}}}}}}}}_{{{{{{{{\rm{re}}}}}}}}}$ is a real matrix given by

29 $${{{{{{{{\bf{B}}}}}}}}}_{{{{{{{{\rm{re}}}}}}}}}=\frac{1}{2}\left({{{{{{{\bf{Re}}}}}}}}\left({{{{{{{{\bf{B}}}}}}}}}_{1}{{{{{{{{\bf{B}}}}}}}}}_{0}^{H}\right){\left({{{{{{{\bf{Re}}}}}}}}\left({{{{{{{{\bf{B}}}}}}}}}_{0}{{{{{{{{\bf{B}}}}}}}}}_{0}^{H}\right)\right)}^{-1}+{{{{{{{\bf{Re}}}}}}}}\left({{{{{{{{\bf{B}}}}}}}}}_{1}{{{{{{{{\bf{B}}}}}}}}}_{1}^{H}\right){\left({{{{{{{\bf{Re}}}}}}}}\left({{{{{{{{\bf{B}}}}}}}}}_{0}{{{{{{{{\bf{B}}}}}}}}}_{1}^{H}\right)\right)}^{-1}\right)$$

with ${{{{{{{\bf{Re}}}}}}}}(\bullet )$ denoting the real part of a complex quantity. For implementation purposes, it is convenient to rewrite matrices **B**_0_ and **B**_1_ in Eqs. (22) and (23), respectively, as

30 $${{{{{{{{\bf{B}}}}}}}}}_{0}=\left[\begin{array}{c}\left(\left\{\begin{array}{c}{z}_{1}^{0}\\ \vdots \\ {z}_{1}^{n}\end{array}\right\}\otimes {{{{{{{\bf{H}}}}}}}}({z}_{1})-\left\{\begin{array}{c}{z}_{0}^{0}\\ \vdots \\ {z}_{0}^{n}\end{array}\right\}\otimes {{{{{{{\bf{H}}}}}}}}({z}_{0})\right)\cdots \left(\left\{\begin{array}{c}{z}_{{N}_{f}}^{0}\\ \vdots \\ {z}_{{N}_{f}}^{n}\end{array}\right\} \otimes {{{{{{{\bf{H}}}}}}}}({z}_{{N}_{f}}) - \left\{\begin{array}{c}{z}_{{N}_{f}-1}^{0}\\ \vdots \\ {z}_{{N}_{f}-1}^{n}\end{array}\right\}\otimes {{{{{{{\bf{H}}}}}}}}({z}_{{N}_{f}-1})\right)\end{array}\right]$$

and

31 $${{{{{{{{\bf{B}}}}}}}}}_{1}=\left[\begin{array}{c}\left(\left\{\begin{array}{c}{z}_{1}^{1}\\ \vdots \\ {z}_{1}^{n+1}\end{array}\right\}\otimes {{{{{{{\bf{H}}}}}}}}({z}_{1})-\left\{\begin{array}{c}{z}_{0}^{1}\\ \vdots \\ {z}_{0}^{n+1}\end{array}\right\}\otimes {{{{{{{\bf{H}}}}}}}}({z}_{0})\right)\cdots \left(\left\{\begin{array}{c}{z}_{{N}_{f}}^{1}\\ \vdots \\ {z}_{{N}_{f}}^{n+1}\end{array}\right\}\otimes {{{{{{{\bf{H}}}}}}}}({z}_{{N}_{f}})-\left\{\begin{array}{c}{z}_{{N}_{f}-1}^{1}\\ \vdots \\ {z}_{{N}_{f}-1}^{n+1}\end{array}\right\}\otimes {{{{{{{\bf{H}}}}}}}}({z}_{{N}_{f}-1})\right)\end{array}\right]$$

where ⊗ denotes the *Kronecker product*. It is well-known, for instance, from refs. ^11,13^, that real-valued eigenvalue problems, as in Eq. (28), yield more stable estimates for the modal properties over the different model orders. In the case of the pCF technique, in particular, this is explained by the fact that the real-valued matrix ${{{{{{{{\bf{B}}}}}}}}}_{{{{{{{{\rm{re}}}}}}}}}$ possesses better numeric condition than its complex counterpart **B**. In modal analysis, a common practice is to plot the so-called stabilization diagram to distinguish the physical modal properties from the numerical ones. An efficient way of constructing a stabilization diagram with the proposed pCF identification technique consists of computing the system matrices **B**_0_ and **B**_1_ for the maximum model order, ${n_{\max}}$, according to Eq. (28).

**Fig. 1:**
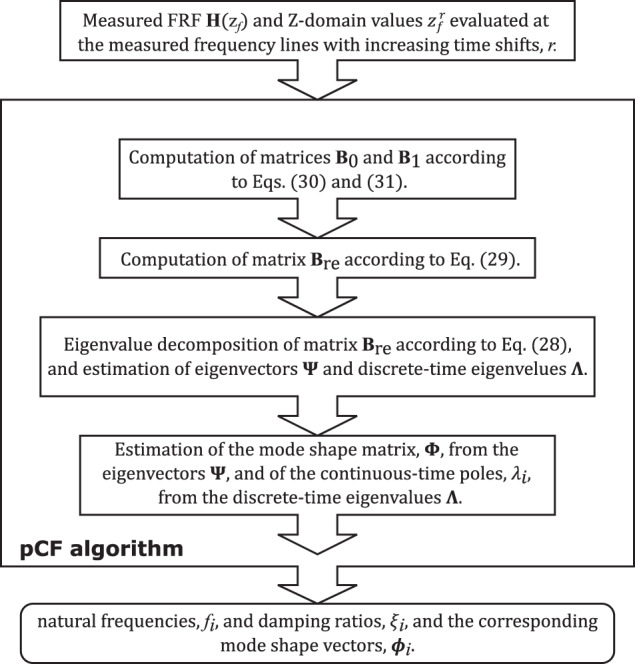
Identification flowchart. Major steps of the identification process with the proposed poly-reference Complex Frequency (pCF) algorithm from the measured Frequency Response Function (FRF).

Once these matrices are computed, the eigenvalues, **Λ**_*n*_, and the eigenvectors, **Ψ**_*n*_, can be computed for increasing model order *n*, i.e., for $n=1,\ldots ,{n_{\max}}$. Since the pCF algorithm uses the full FRF matrix in the computation of system matrices **B**_0_ and **B**_1_, the estimation of the modal properties with such an algorithm might be time-consuming. In order to improve the computational performance of the algorithm, one should consider using the approach driven by the Discrete Fourier Transform described in ref. ^13^. A flowchart illustrating the main steps of the identification process with the **pCF** algorithm is shown in Fig. 1.

## Results

In order to assess its accuracy and robustness, the pCF approach is applied to three application examples, namely, a simulated input-output vibration test of a T-shaped steel structure and output-only vibration tests of a steel specimen and of a 15-storey reinforced concrete building. The analysis performed, as well as the results obtained from such tests, are described as follows.

### Application example 1: Simulated input-output vibration test of a T-shaped structure

**Fig. 2:**
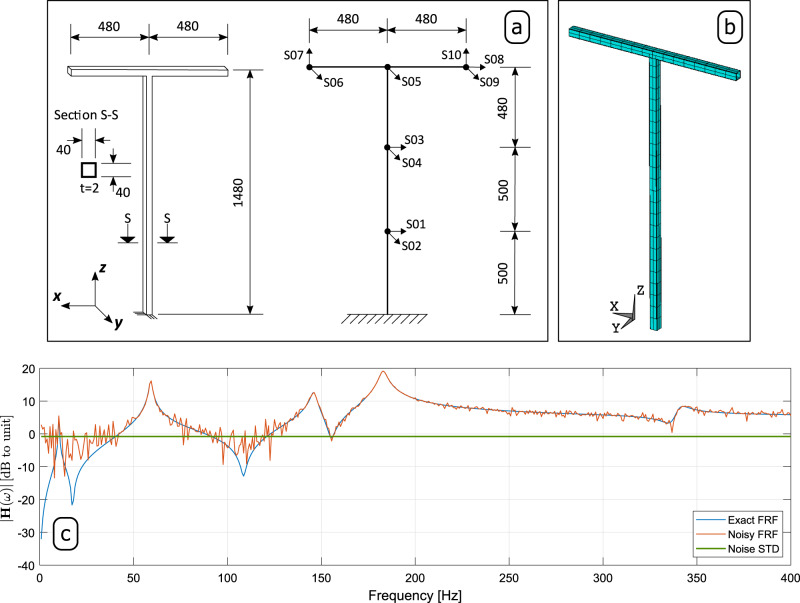
Details of the T-shaped structure and its simulated FRF. **a** Dimensions and measured Degrees of Freedoms, **b** Finite Element model used to generate the Frequency Response Function (FRF) matrix by means of state-space simulation; and **c** element (10,5) of the FRF matrix **H**(*ω*) wherein the blue solid line corresponds to the exact FRF, the red solid line to the FRF contaminated with noise, and the solid green line to the noise STandard Deviation (STD).

The first application example used to demonstrate the robustness of the pCF algorithm consists of a EMA simulated with a Finite Element (FE) model of a T-shaped structure. The dimensions of such structure and the position of the sensors considered in the simulated EMA are shown in Fig. 2a, and the corresponding FE model with a total of 240 Degrees Of Freedom (DOFs) is depicted in Fig. 2b. The FE model comprising 40 beam elements with 6 DOFs per node (e.g., three translations and three rotations) was clamped at the bottom-most node. The exact first 10 eigenfrequencies and damping ratios used in the simulated EMA are summarized in Table 1, and the corresponding exact mode shape vectors are shown in Supplementary Fig. 1 and Supplementary Table 1.

**Table 1:** Exact modal properties computed from the finite element model of the T-shaped structure

Mode	Type	Natural frequency (*f*_*i*_) [Hz]	Damping ratio (*ξ*_*i*_) [%]	Modal mass (*m*_*i*_) [Kg]
1	1st bending mode along X direction (BX1)	10.1882	1.0	3.2187
2	1st bending mode along Y direction (BY1)	10.4626	1.0	3.1178
3	1st torsional mode (T1)	28.8591	1.0	0.1726
4	2nd bending mode along X direction (BX2)	59.1011	1.0	1.1040
5	2nd bending mode along Y direction (BY2)	92.8089	1.0	0.1874
6	3rd bending mode along X direction (BX3)	145.9538	1.0	0.2968
7	1st bending mode along Z direction (BZ1)	182.8250	1.0	0.0659
8	3rd bending mode along X direction (BY3)	250.1182	1.0	0.0815
9	4th bending mode along X direction (BX4)	318.4404	1.0	0.0524
10	4th bending mode along Y direction (BY4)	340.7061	1.0	0.0704

The structural damping was modeled as the special case of proportional damping by setting the damping coefficients of all modes equal to 1%. In order to simulate the vibration responses, the T-structure was excited independently at DOFs S01, S02, S03, S04, S05, S06, S07 and S08, shown in Fig. 2a with a white Gaussian noise. The simulated responses were measured in acceleration at all DOFs, yielding a FRF matrix with 10 rows and 8 columns. Assuming that the two beams are rigid along their neutral axes, i.e., that there is no axial deformation, the measured outputs are enough to yield the modal configurations of the first 10 vibration modes of the T-structure. The exact FRF matrix was computed by means of the frequency-domain state-space formulation in the frequency range of 0–400 Hz. The resulting FRF matrix was evaluated at a total of 1024 frequency lines with a resolution of 390.625 mHz.

Afterward, the simulated FRF containing only the contribution of the first 10 vibration modes was contaminated with white noise sequences with three different values for the standard deviation to mimic FRFs estimated in real vibration tests. Noise standard deviations of 1.0%, 0.01% and 0.0001% were used to yield noise-contaminated FRFs with Signal-to-Noise Ratios (SNRs) of 20, 40 and 60 dB, respectively. This was achieved by adding a complex random number to the FRF at each frequency line. This number was computed such that its amplitude was given as a random number of a normal distribution with zero mean and the considered standard deviation times the maximum absolute value of the FRF, and its phase was a uniform random number between 0 and 2*π*. In the following, the identification results obtained with the pLSCF and pCF techniques from the noise-contaminated FRF with an SNR of 20 dB are presented. The results from the FRFs contaminated with SNRs of 40 and 60 dB are shown in Supplementary Figs. 2 and 3. Figure 2c shows the element (10,5) of the noise-contaminated FRF matrix with an SNR of 20 dB, its corresponding exact counterpart and its noise standard deviation.

**Fig. 3:**
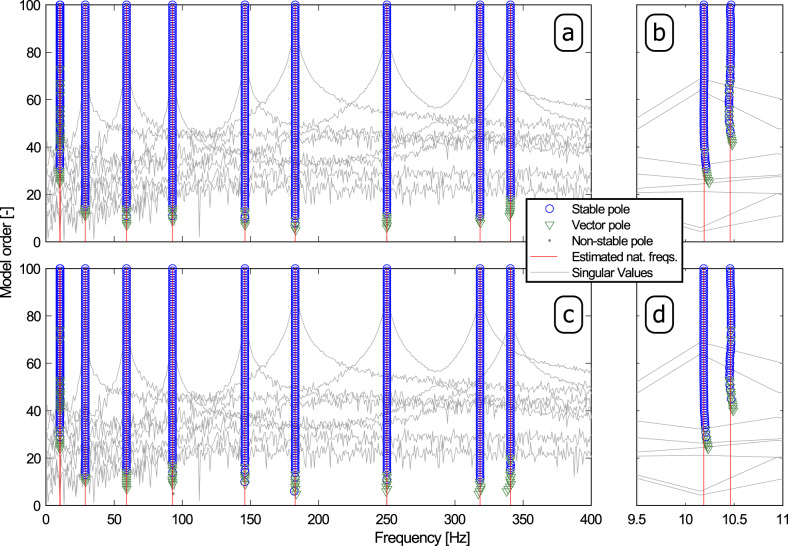
Diagrams constructed from the noise contamineted FRF of the T-shaped structure with a SNR of 20 dB. **a**, **b** Poly-reference Least Squares Complex Frequency (pLSCF) and **c**, **d** poly-reference Complex Frequency (pCF) stabilization plots constructed from the noise contaminated Frequency Response Function (FRF) with a Signal-to-Noise Ratio (SNR) of 20 dB by identifying models with order, *n*, ranging from 1 to 100. **b**, **d** Details of the two closely spaced modes around 10.5 Hz.

Next, the pLSCF and pCF methods were applied to the noise-contaminated FRF matrices. The pLSCF technique is known for being a very accurate and robust frequency-domain identification algorithm. Hence it is deemed nowadays as a standard modal identification approach by the modal analysis community and is commercialized in several modal identification software. This accuracy and robustness allow for the creation of clear stabilization diagrams that facilitate the identification of the physical modal properties. Therefore, the pLSCF algorithm is used as a reference in the three application examples presented in this paper. In order to sort out the physical modal properties from the numerical ones, stabilization diagrams were constructed with both techniques by identifying models with order, *n*, ranging from 1 to 100. The stabilization diagrams constructed with pLSCF and pCF are shown in Fig. 3a, b and in Fig. 3c, d, respectively, wherein the vertical solid red lines indicate the natural frequencies automatically identified with the hierarchical clustering algorithm described in refs. ^20–22^. Figure 3a, c presents the identification results in the frequency range of 0 to 400 Hz, while Fig. 3b, d shows the details of the closely spaced modes around 10.4 Hz.

**Fig. 4:**
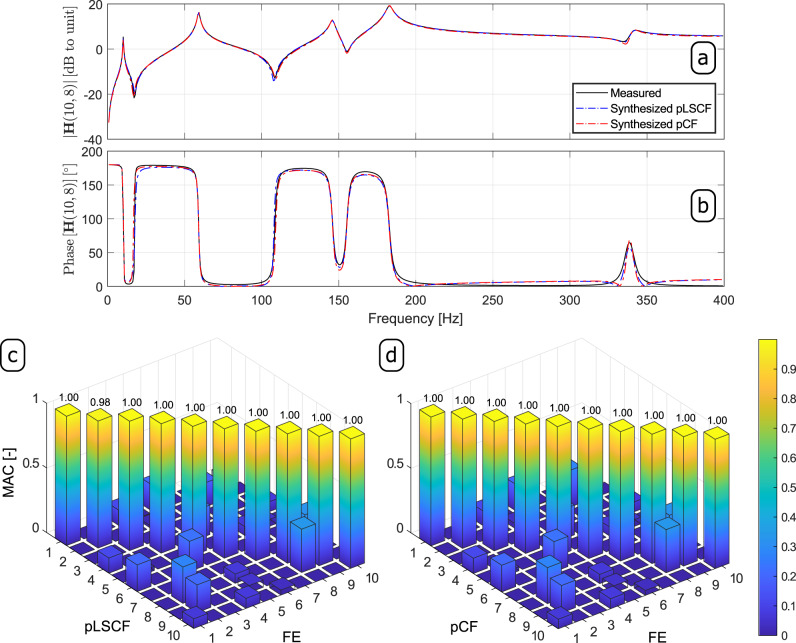
Results obtained from the vibration data of the T-shaped structure. Measured and synthesized Frequency Response Function (FRF) matrix **H**(*ω*): **a** magnitude and **b** phase of the element (10,8) of the FRF matrix; **c** Modal Assurance Criterion (MAC) between the Finite Element (FE) modal vectors and those estimated with the poly-reference Least Squares Complex Frequency (pLSCF) technique, and **d** MAC between the FE modal vectors and those estimated with proposed poly-reference Complex Frequency (pCF) approach from the noise-contaminated FRF with a Signa-to-Noise Ratio of 20 dB.

Comparing the results shown in Fig. 3a, b to those depicted in Fig. 3c, d, it becomes clear that the pCF performs as robust as the pLSCF algorithm. The natural frequencies, damping ratios and modal participation factor vectors automatically identified with the aforementioned hierarchical cluster algorithm from the set of estimates obtained with both methods were subsequently used to compute the mode shape vectors by means of the so-called Least Squares Frequency Domain (LSFD) algorithm^21,25,30^. The computation of the mode shape vectors with such an algorithm was carried out with no upper and lower residuals since there is no influence of out-of-band modes in the frequency range of interest. Finally, all the estimated modal properties were used to synthesize the FRF matrix. In Fig. 4, elements (10,8) of the FRF matrices synthesized from the pLSCF and pCF estimates are compared to their exact counterparts in terms of phase (Fig. 4a) and magnitude (Fig. 4b).

By comparing the results shown in such figures, it is clear that the proposed pCF technique performs as robustly as the pLSCF approach in terms of input-output broad band modal identification, even when dealing with very closely spaced modes. With regard to the mode shape vector estimates, the proposed pCF provided slightly better accuracy than the pLSCF technique, particularly in the estimation of the second modal vector. This is verified in Fig. 4c, d, where the Modal Assurance Criterium (MAC) between the FE mode shape vectors and their estimates from both techniques are shown. The pCF also performed as robustly as the pLSCF approach when applied to the noisy FRFs with SNRs of 40 and 60 dB, as shown in Supplementary Figs. 2 and 3. The natural frequencies and damping ratios estimated with pLSCF and pCF techniques are summarized in Table 2. In this table, it is also shown the accuracy of the estimates provided by both techniques in terms of relative error. By comparing these results, one verifies that the pCF provided estimates for the natural frequencies and damping ratios that are in good agreement with their exact counterparts. Although the relative error of the pCF estimates for the damping ratios are slightly higher than those of the pLSCF technique, they are still reasonably close to the exact ones.

**Table 2:** Results obtained with the poly-reference Least Squares Complex Frequency (pLSCF) and the proposed poly-reference Complex Frequency (pCF) for the T-Structure

Mode	pLSCF estimates				pCF estimates			
	${\hat{f}}_{i}$	Rel. error	${\hat{\xi }}_{i}$	Rel. error	${\hat{f}}_{i}$	Rel. error	${\hat{\xi }}_{i}$	Rel. error
	(Hz)	(% × 10^3^)	(%)	(%)	(Hz)	(% × 10^3^)	(%)	(%)
1	10.192	33.46	0.94	52.78	10.189	3.71	0.92	54.15
2	10.459	36.35	0.78	61.11	10.463	0.10	0.62	68.95
3	28.861	5.97	0.96	51.90	28.857	8.20	0.95	52.35
4	59.105	7.14	0.98	51.14	59.098	5.43	0.94	53.01
5	92.806	3.40	0.99	50.33	92.802	7.03	0.89	55.71
6	145.953	0.88	1.00	50.10	145.940	9.30	0.86	56.99
7	182.822	1.42	0.99	50.29	182.821	2.22	0.90	55.07
8	250.114	1.72	1.00	50.24	250.104	5.72	0.86	57.25
9	318.448	2.46	1.00	50.24	318.419	6.62	0.84	58.14
10	340.720	4.14	0.99	50.39	340.676	8.83	0.71	64.57

### Application examples 2: Output-only vibration test of a platform specimen

**Fig. 5:**
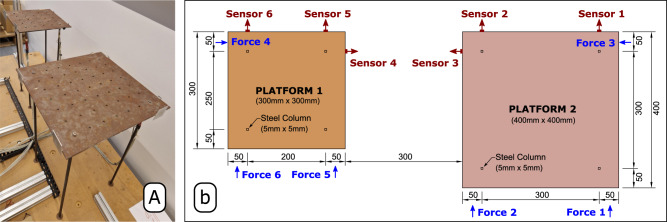
Setup adopted in the vibration test of the steel platform specimens. **a** Photo of the real platform specimen, **b** dimensions (in millimeters) of the top-site of the steel platform specimens, excitation forces, sensors' positions and measurement directions.

The second application example consists of the OMA of two independent steel platform specimens shown in Fig. 5a.

The specimens were used in a previous study to investigate the coupled dynamic behavior of offshore oil platforms when they are connected by a bridge^31,32^. Each platform comprises one 5 mm-thick steel plate and four steel columns with squared cross sections placed 50 mm away from the edges of the top plates. The top-site steel plates are squared with dimensions of 300 × 300 and 400 × 400 mm, as indicated in Fig. 5b. The steel columns, which are clamped to the steel plates at the top and to a stiff wooden box at the bottom, are 600 mm high and have squared cross sections 5 mm wide. The vibration responses in acceleration were measured at a total of 6 DOFs, three of which were measured on the platform with a smaller plate and three on the bigger one, as also indicated in Fig. 5b.

The two steel platforms were randomly excited at six different positions (indicated by the blue arrows in Fig. 5b) by a pneumatic actuator whose airflow was instantaneously adjusted by valves actively controlled by an algorithm. The excitation signals used to control each valve were independently generated by the algorithm with flat spectral densities ranging from 2 up to 20 Hz to secure a proper excitation of the first five modes. The vibration responses due to the airflow excitation were measured in the acceleration with HBK accelerometers (piezoelectric CCLD with TEDS, with a sensitivity of 100 mV/g) with a sampling rate of 1652.9 Hz^27,33^. A total of 164,993 time samples were acquired in the output-only vibration test, which corresponds to a measurement duration of approximately 1 min and 40 s. Afterward, the acceleration time series underwent a signal processing step to estimate the so-called Half Spectrum (HS) matrix ${{{{{{{{\bf{S}}}}}}}}}_{yy}^{+}(\omega )$.

**Fig. 6:**
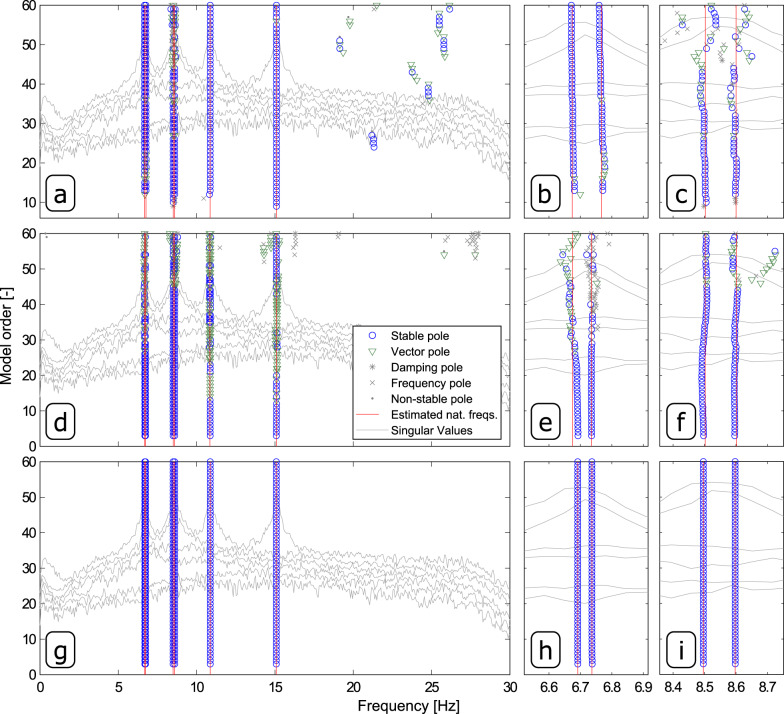
Stabilization diagrams constructed to estimate the modal properties of the platform specimens. Results obtained with Ibrahim Time Domain (ITD) (**a**–**c**), poly-reference Least Squares Complex Frequency (pLSCF) (**d**–**f**) and poly-reference Complex Frequency (pCF) (**g**–**i**) from the vibration responses of the two steel platform specimens by identifying models with order ranging from 1 to 60, and details of the closed spaced modes automatically identified around 6.7 Hz (**b**, **e**, **h**) and 8.5 Hz (**c**, **f**, **i**).

To achieve this goal, they were de-trended, filtered with an 8th order lowpass Chebyshev Type I filter and resampled with a sampling frequency of 68.8705 Hz. Finally, the HS matrix was computed by taking the Fourier transform of the positive part of the Correlation Function (CF) matrix estimated with 1024 time lags. The estimation of the HS matrix was carried out by applying an exponential window with a decay rate of 95% to the positive part of the CF function to minimize the leakage effect on the estimated HS. Once computed, the HS matrix with dimension 6 × 6 × 513 was used input data both by the pCF and the pLSCF algorithm and the CF matrix by the ITD technique. In order to facilitate the identification of the physical modes of the platforms, stabilization diagrams were constructed with the pCF and the pLSCF by identifying models with order ranging from 1 to 60 and with the ITD technique by varying the dimensions of the system matrices from 1 to 60. Figure 6 shows the stabilization diagrams constructed with ITD, pLSCF and pCF techniques. The identification of the physical modes in such diagrams was again carried out by means of a hierarchical cluster algorithm.

**Fig. 7:**
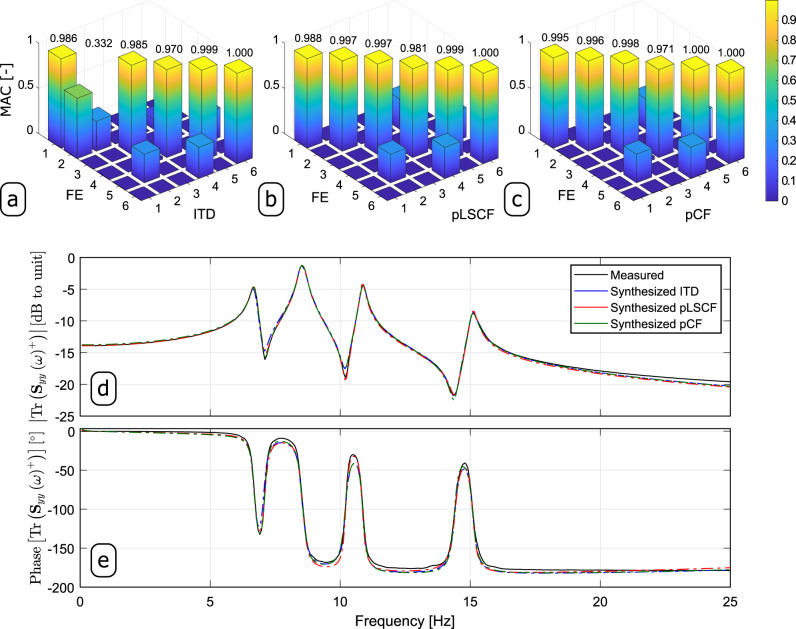
Results obtained from the vibration data of the platform specimens. **a** Modal Assurance Criterion (MAC) between the Finite Element (FE) and Ibrahim Time Domain (ITD) modal vectors. **b** MAC between the FE modal vectors and the poly-reference Least Squares Complex Frequency (pLSCF) modal vectors. **c** MAC between the FE and poly-reference Complex Frequency (pCF) modal vectors. Comparison in terms of **d** magnitude and **e** phase of the Trace (Tr) of the measured Half Spectrum (HS) matrix $\left({{{{{{{{\bf{S}}}}}}}}}_{yy}^{+}(\omega )\right)$ with the Traces of the HS matrices synthesized with the ITD, pLSCF and pCF estimates.

These modes are indicated by the vertical solid red lines in Fig. 6. Comparing the results shown in Fig. 6, it is obvious that the pCF provided clearer stabilization plots whose stable poles matched the peaks of the spectral density singular values (estimated with the classic Frequency Domain Decomposition technique^19^) shown in the background solely for comparison purposes. The estimates for the mode shape vectors computed with ITD, pLSCF and pCF are compared in terms of MAC with the estimates from an FE model of the platforms in Fig. 7. In these correlation analyses, the results obtained with the FE model of the two platforms shown in the Supplementary Fig. 4 are used as reference modal properties. As seen in Fig. 7a–c, the pCF estimates for the first 6 six vibration mode shapes are slightly better correlated with those from the FE model than those computed with ITD and pLSCF.

Aiming at assessing the accuracy of the modal parameter estimation obtained with the three identification techniques, the traces of the synthesized HS matrices are compared to that of the measured HS in terms of magnitude (Fig. 7d) and phase (Fig. 7e). Comparing the traces synthesized from the estimates of each technique to their measured counterpart in such figures, it is clear that the pCF provided very accurate estimates for the modal properties of the platform specimens. The estimates for the natural frequencies and damping coefficients computed with ITD, pLSCF and pCF are summarized in Table 3, and the corresponding mode shape vectors are shown in Supplementary Figs. 5–7. The mode shapes shown in such figures were plotted with the aid of slave DOFs under the assumption that the top plates on both platforms behave as rigid bodies. By comparing the results shown in Table 3 and Supplementary Figs. 5–7, one verifies that the pCF approach provided estimates for the modal properties of the platform specimens that match those obtained with the ITD and pLSCF approach.

**Table 3:** Natural frequencies $({\hat{f}}_{i})$ and damping coefficients $({\hat{\xi }}_{i})$ of the platform specimen estimated with Ibrahim Time Domain (ITD), poly-reference Least Squares Complex Frequency (pLSCF) and poly-reference Complex Frequency (pCF)

Mode	ITD		pLSCF		pCF	
	${\hat{f}}_{i}$ (Hz)	${\hat{\xi }}_{i}$ (%)	${\hat{f}}_{i}$ (Hz)	${\hat{\xi }}_{i}$ (%)	${\hat{f}}_{i}$ (Hz)	${\hat{\xi }}_{i}$ (%)
1	6.6702	0.883	6.6952	0.949	6.6912	0.711
2	6.7535	0.552	6.7365	0.468	6.7368	0.497
3	8.4829	0.554	8.5022	0.514	8.4965	0.577
4	8.5899	0.737	8.6003	0.578	8.5974	0.594
5	10.8611	0.382	10.8514	0.267	10.8642	0.445
6	15.1008	0.507	15.0980	0.431	15.0873	0.555

### Application examples 3: Output-only vibration test of a 15-storey reinforced concrete building

**Fig. 8:**
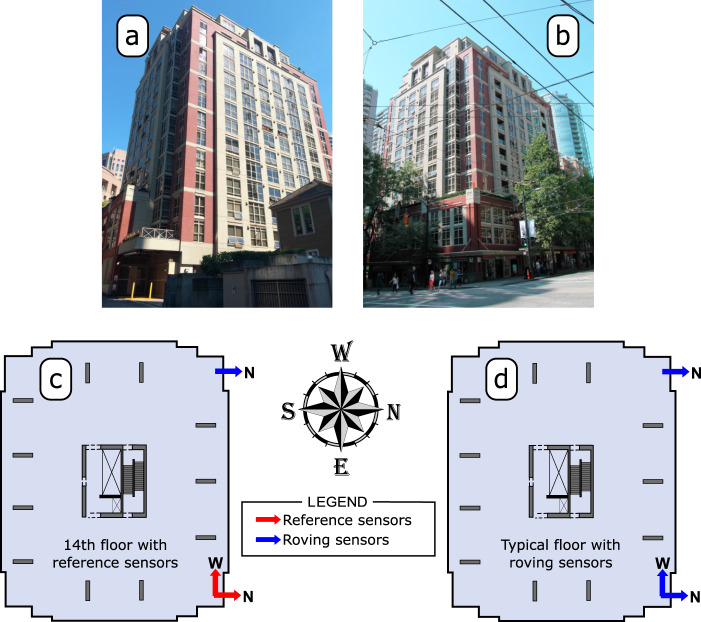
Heritage Court Tower in Vancouver, Canada. **a** Northern and **b** eastern facades of the 15-storey reinforced concrete building, **c** detail of the 14th floor where the reference sensors were placed and **d** typical sensors’ locations and directions on every second floor.

The third application example comprises the vibration responses of the Heritage Court Tower (HCT) (depicted in Fig. 8a, b), which are used as real-life OMA examples. The HCT is a relatively regular 15-storey reinforced concrete shear core building located at the corner of Hamilton and Robson in Vancouver, British Columbia, Canada. Figure 8a shows the northern and Fig. 8b the eastern facade of the building. The HCT vibration measurements are a well-known set of data in OMA literature. It is also well-known in the modal analysis community for being a difficult multi-dataset application example because is based only on two reference sensors^37^. The original publication of the ambient vibration test of the HCT is found in ref. ^34^, and the full OMA of the data acquired in such test was presented in the same year of this publication in ref. ^16^. The HTC test is also one of the examples in ref. ^19^, and the data is a part of the associated Matlab toolbox^35^.

A detailed description of the ambient vibration test of the HCT is found in APPENDIX B of ref. ^19^. The HCT data comprise a total of four datasets. The first contains the data of only six measurement channels, and the remaining datasets gather the data of eight channels. Eight force balance accelerometers were available for the ambient vibration test, two of which were used as reference, whereas the remaining ones were used as roving sensors. The former were mounted on the 14th (and second topmost) floor and kept at the same position throughout the test. The latter, on the other hand, were conveniently moved from dataset to dataset so that the vibration responses of a total of 30 DOFs were measured along the height of the structure. Acceleration responses of three DOFs were measured on every second floor, beginning from the roof of the uppermost penthouse down to the second floor. Figure 8c shows a detail of the 14th floor where the reference sensors were mounted, and Fig. 8d depicts the typical sensors’ locations and measurement directions on every second floor. All datasets were acquired with a sampling rate of 40 Hz for approximately 5 min and 28 s. In order to estimate the first eight vibration modes, all datasets were filtered with an eight-order Chebyshev type I lowpass filter with a cutoff frequency of 8 Hz and resampled with a sampling rate of 10 Hz.

Once conveniently processed, the measured acceleration responses were used to estimate the HS matrix, ${{{{{{{{\bf{S}}}}}}}}}_{yy}^{+}(\omega )$, which replaces the FRFs in the case of OMA. The non-parametric estimation of HS from vibration response measurements is described in several publications, for instance, in refs. ^12,19,21^. The benefit of using the HS in OMA is that it can be parameterized in the same way as the FRF^16,19,36^, and, therefore, it can be used as input data by all the frequency-domain parametric modal identification techniques initially designed for EMA. The estimation of the HS was basically carried out in three steps.

First, the positive CFs were estimated from the filtered acceleration responses for a total of 1024 time lags in order to yield accurate HS estimates. Afterward, an exponential window with a decay rate of 99.999% was applied to the resulting CF matrices in order to minimize the leakage and the influence of the noise tail. Finally, the HS of each dataset was computed by taking the Fourier Transform of the windowed CFs. This yielded HS matrices with a total of 513 frequency lines with a frequency resolution of 39.216 mHz.

**Fig. 9:**
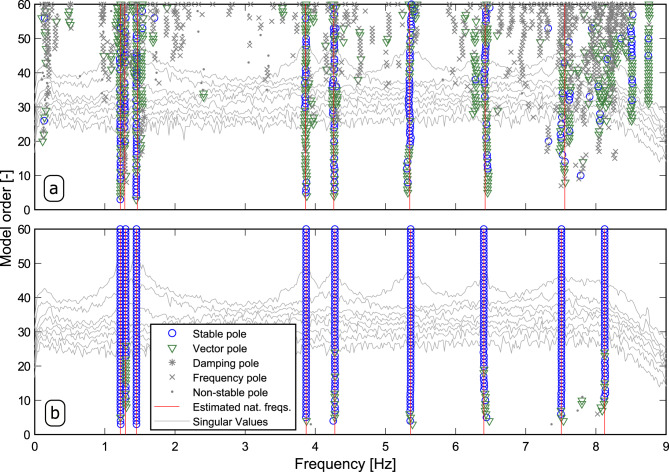
Stabilization diagrams constructed from the vibration responses of the HCT stored in dataset 4. **a** Poly-reference Least Squares Complex Frequency (pLSCF) and **b** poly-reference Complex Frequency (pCF) results obtained from the vibration responses of the Heritage Court Tower (HTC) recorded in dataset 4 by identifying models with order, *n*, ranging from 1 to 60.

The pLSCF and pCF techniques were then applied to the HS matrix estimated from dataset four, which is regarded as the most difficult among all the acquired datasets due to the weak ambient excitation at the time of the test. Stabilization diagrams were constructed with both techniques from the vibration responses of all four datasets to distinguish the physical modal properties from the numerical ones. Figure 9a, b shows the diagrams constructed from the vibration responses of the fourth dataset by identifying models with order, *n*, ranging from 1 to 60, wherein the vertical solid red lines indicate the identified natural frequencies. The light gray lines in the background of such figures are the singular values obtained by computing the singular value decomposition of the spectral density matrix^19^ at each frequency line. These lines are plotted merely to confirm that the identified natural frequencies are physical. The stability criteria used in the diagrams of Fig. 9a, b were: 1% for natural frequencies, 7.5% for damping ratios and 5% for mode shape vectors.

By comparing such figures, it is noticed that a much clearer diagram was obtained with the proposed pCF algorithm. The pCF method has the interesting ability of providing negative damping ratio estimates for the non-physical poles, which makes it easy to exclude them from the set of estimated modal properties before plotting them. Another distinguishing characteristic of the pCF is that the physical modal properties tend to remain fairly stable for increasing model orders. Similarly to the previous application example, the natural frequencies, damping ratios and mode shape vectors identified from dataset 1 with both methods were then used to compute the modal participation factor vectors by means of the LSFD algorithm^25^. The computation of such vectors was carried out with the upper and lower residuals to minimize the influence of the out-of-band vibration modes.

**Fig. 10:**
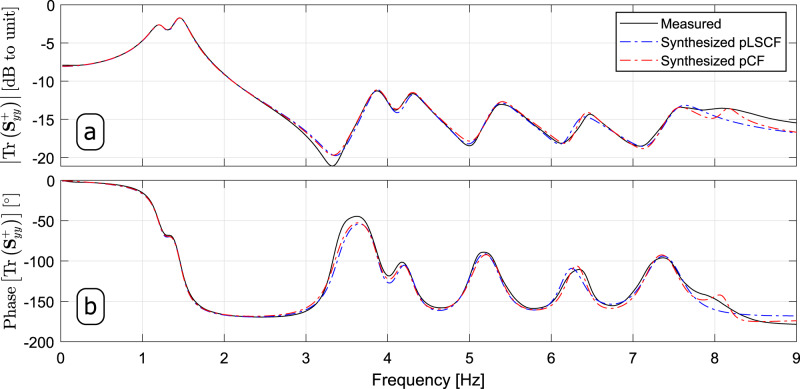
Half spectral matrix of dataset 4. Comparison of the Trace (Tr) of the measured Half Spectral (HS) matrix in terms of **a** magnitude and **b** phase with trace of the HS matrix synthesized with the estimates obtained with the poly-reference Least Squares Complex Frequency (pLSCF) and with the poly-reference Complex Frequency (pCF).

Once the modal participation factor vectors were estimated, they were used to synthesize the HS matrix (${{{{{{{{\bf{S}}}}}}}}}_{yy}^{+}(\omega )$) using the modal model with upper and lower residual terms^21,23^. In Fig. 10, the traces of the HS matrices synthesized with the pLSCF and pCF estimates are compared to the trace of the measured HS matrix both in terms of magnitude (Fig. 10a) and phase (Fig. 10b). Comparing the results shown in such figures, it becomes clear that the pCF technique provided clearer estimates for the modal properties of the HCT than the pLSCF approach. Similar results were obtained from the remainder of the datasets, as summarized in Table 4. These results show that the pCF approach provided estimates for the modal properties of the HCT that are fairly similar to those of the pLSCF approach. The Stabilization diagrams constructed from datasets 1, 2 and 3 are shown in Supplementary Figs. 8a, b, Supplementary Figs. 9a, b, and Supplementary Figs. 10a, b, respectively, where the (a) panels in these figures depict diagrams constructed with the pLSCF technique and the (b) ones show the diagrams created with the proposed pCF. The results synthesized by these diagrams corroborate the tendency of the pCF method to provide estimates for the modal properties in a more robust fashion than the pLSCF approach, regardless of the model order utilized in the identification process.

**Table 4:** Poly-reference Least Squares Complex Frequency (pLSCF) and poly-reference Complex Frequency (pCF) estimates obtained from all datasets acquired in the vibration test of the Heritage Court Tower

Technique	Mode	Dataset 1		Dataset 2		Dataset 3		Dataset 4		Average	
		${\hat{f}}_{i}$ (Hz)	${\hat{\xi }}_{i}$ (%)	${\hat{f}}_{i}$ (Hz)	${\hat{\xi }}_{i}$ (%)	${\hat{f}}_{i}$ (Hz)	${\hat{\xi }}_{i}$ (%)	${\hat{f}}_{i}$ (Hz)	${\hat{\xi }}_{i}$ (%)	${\mu }_{{\hat{f}}_{i}}$ (Hz)	${\mu }_{{\hat{\xi }}_{i}}$ (%)
pLSCF	1	1.227	1.068	1.231	1.897	1.234	1.087	1.220	1.715	1.228	1.442
	2	1.289	1.186	1.289	1.270	1.290	1.043	1.284	1.760	1.288	1.315
	3	1.448	1.487	1.461	1.750	1.456	0.820	1.464	0.960	1.457	1.254
	4	3.862	1.512	3.880	1.275	3.875	1.305	3.861	1.053	3.869	1.286
	5	4.249	1.186	4.254	1.625	4.260	1.209	4.264	1.230	4.257	1.313
	6	5.396	1.442	5.406	0.868	5.339	0.711	5.347	1.728	5.372	1.187
	7	6.424	1.138	6.432	0.955	6.369	1.065	6.420	1.298	6.411	1.114
	8	–	–	–	–	–	–	7.557	0.802	–	–
pCF	1	1.226	0.937	1.230	1.628	1.233	0.957	1.221	1.645	1.227	1.292
	2	1.288	1.114	1.287	1.088	1.291	1.003	1.297	1.074	1.291	1.070
	3	1.448	1.237	1.455	1.276	1.457	0.741	1.450	0.935	1.452	1.047
	4	3.866	1.285	3.875	1.229	3.873	1.223	3.869	1.135	3.871	1.218
	5	4.247	1.116	4.260	1.445	4.258	1.236	4.279	0.946	4.261	1.186
	6	5.382	1.486	5.397	1.171	5.346	2.054	5.358	1.546	5.371	1.564
	7	6.425	0.636	6.426	0.835	6.381	1.054	6.403	0.783	6.409	0.827
	8	7.581	0.933	7.582	0.518	7.563	0.183	7.506	0.792	7.558	0.607

Moreover, the results summarized in Table 4 also show that the proposed pCF algorithm provided estimates for the first eight modes of the HCT, while only the first seven vibration modes were identified with the pLSCF technique. The global mode shape configurations of the HCT estimated with the pLSCF method are shown in Supplementary Fig. 11, whereas the global mode shapes estimated with the pCF approach are illustrated in Supplementary Fig. 12. These global mode shapes were estimated by following the classic merging approach described, for instance, in refs. ^21,37^, i.e., by merging the mode shape parts with the aid of reference sensors, which are common to all datasets. Comparing the mode shape configurations shown in such figures, it is observed that the pCF method provided mode shape estimates that are very similar to those estimated with the pLSCF approach.

## Conclusion

A poly-reference frequency-domain modal identification technique is proposed in this paper. Such an approach is formulated using a frequency-domain function (e.g., the FRF or HS) in Z-domain modal model. The underlying idea behind its formulation is to estimate the modal properties by comparing the frequency-domain function evaluated at two neighboring frequency lines. By following this strategy, two system matrices can be computed solely from the measured frequency-domain vibration data and the Z-domain variables. Once such matrices are computed, they are used to formulate an eigenvalue problem that, in turn, is solved to compute the modal properties. The method yields very clean stabilization diagrams, facilitating the task of identifying the physical modal properties of the tested structural systems. This robustness follows from the fact that the pCF algorithm tends to yield numerical poles with negative damping ratios, facilitating their removal from the stabilization diagrams and, thereby, the identification of the physical modal properties.

The proposed technique was compared to the commercial pLSCF method, which is nowadays regarded as a standard in frequency-domain modal parameter estimation due to its ability of providing clear stabilization diagrams and accurate estimates for the modal properties. This comparison was carried out by means of three application examples: a simulated EMA and two real-life OMA. In the first application example, the pCF provided very clear stabilization diagrams even in the presence of very close-spaced vibration modes and highly noise-contaminated FRFs. In the last two real-life output-only examples, the proposed method provided much clearer diagrams than the classic pLSCF technique, which allowed for a robust and accurate identification of the physical dynamic properties of the tested structural systems.

### Supplementary information

Supplementary information

## Data Availability

The vibration data of the HCT used as real life application example can be downloaded at https://brincker-monitoring.com/oma-toolbox/. The vibration response data of the steel platform specimens used in the second application example is available from the first author upon request. The FRF data used in the first application example can be simulated by means of the frequency-domain state-space model with the properties shown in Table 1 and Supplementary Table 1.

## Code availability

The application examples presented in this paper were elaborated with the signal processing tools found in Prof. R.B.’s OMA toolbox, which can be downloaded at https://brincker-monitoring.com/oma-toolbox/. Regarding the code related to pCF methodology, the authors are currently pursuing IP rights. Once this process is complete, the code will be made available upon reasonable request.
