# Supplementary material for: A robust poly-reference frequency-domain identification method to extract dynamic properties from vibration data
Source: Commun Eng. 2023 Oct 17;2:73. doi: 10.1038/s44172-023-00122-y (PMC11316028; doi:10.1038/s44172-023-00122-y)
Supplement: Supplementary file 1 — Supplementary information [file 44172_2023_122_MOESM1_ESM.pdf]

Supplementary Information

I. Supplementary Figures

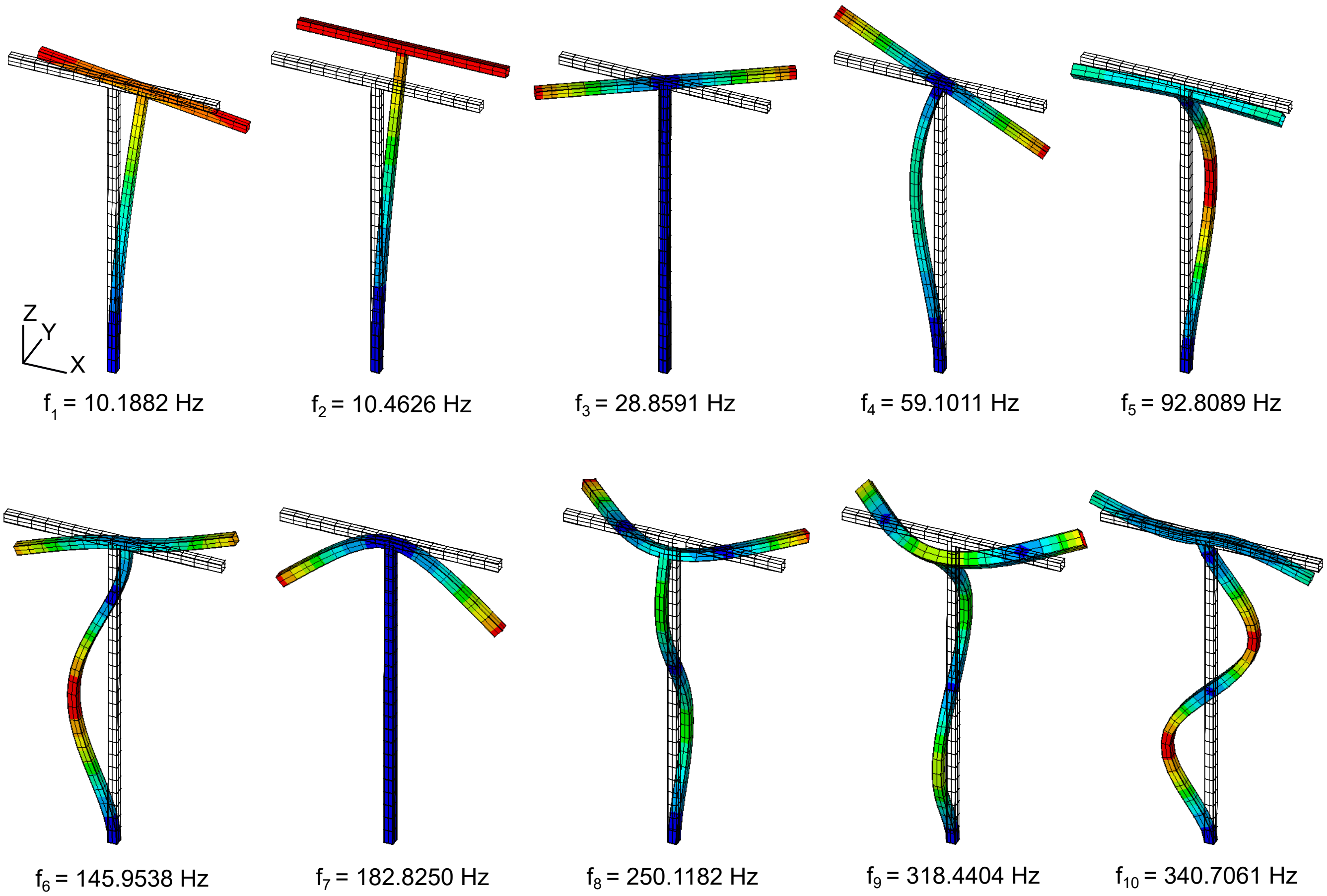

Supplemetary Fig. 1. Simulated modal properties of the T-shaped structure. Exact natural frequencies and mode shapes of the T-shaped structure obtained with its Finite Element model.

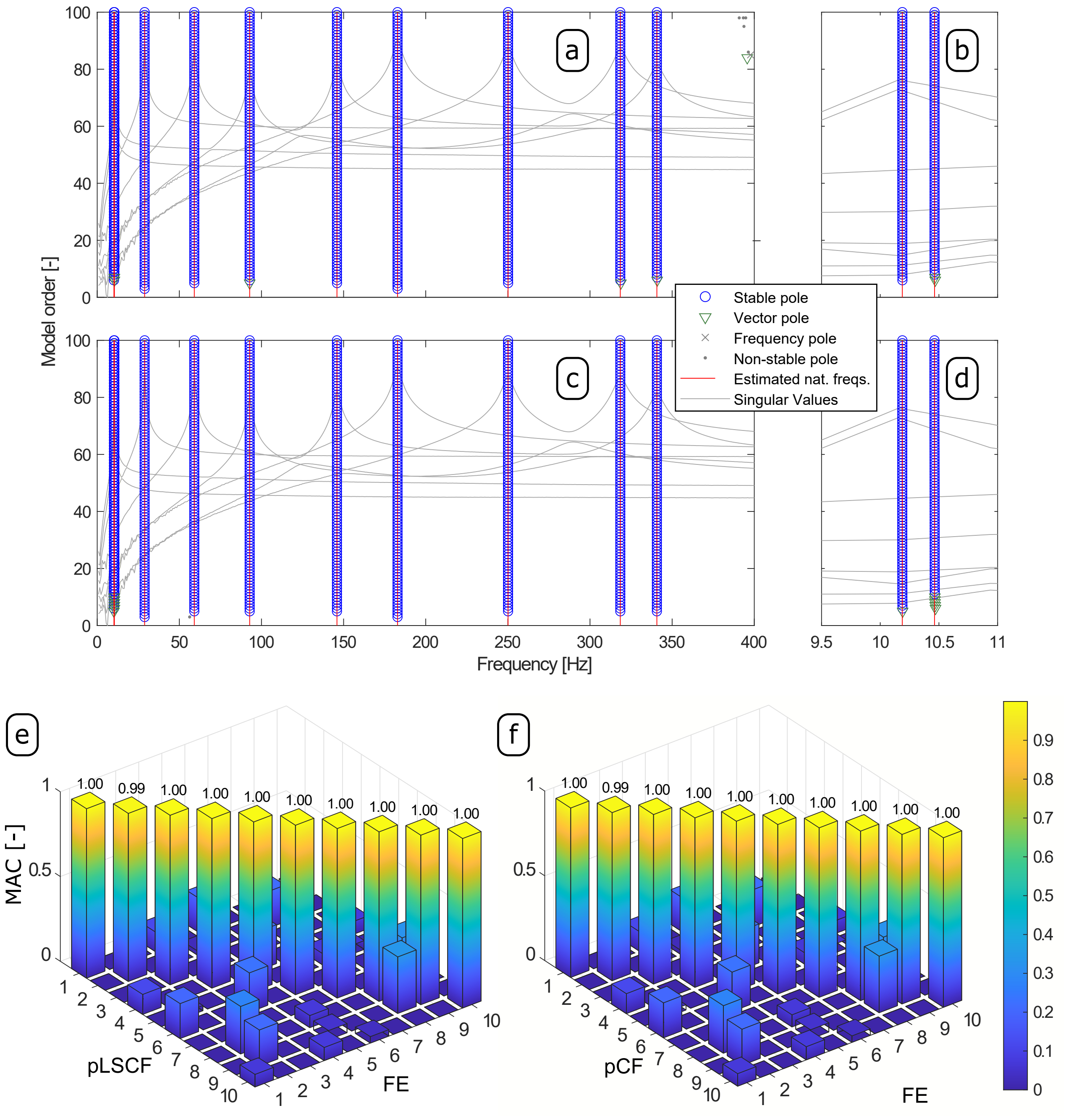

Supplemetary Fig. 2. Results extracted from the simulated FRF of the T-shaped structure contaminated with a SNR of 40dB. (**a**, **b**) poly-reference Least Squares Complex Frequency (pLSCF) and (**c**, **d**) poly-reference Complex Frequency (pCF) stabilization plots constructed from the Frequency Response Function (FRF) contaminated with noise with a Signal-to-Noise Ratio (SNR) of 40dB by identifying models with order, *n*, ranging from 1 to 100. (**b**, **d**) details of the two closely spaced modes around 10.5 Hz. (**c**) Modal Assurance Criterion (MAC) between the Finite Element (FE) modal vectors and those estimated with the pLSCF technique. (**d**) MAC between the FE modal vectors and those estimated with the proposed pCF approach.

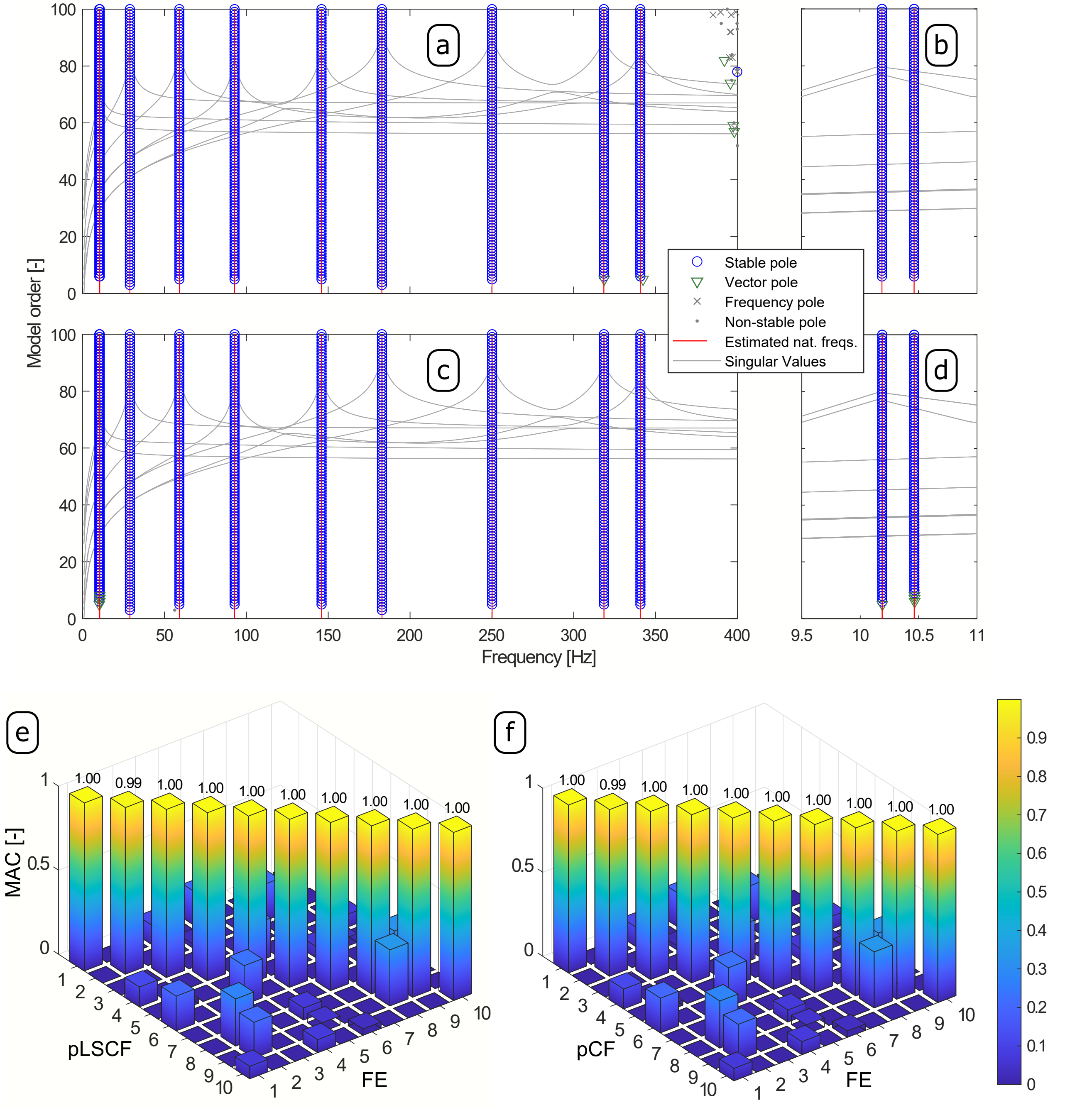

Supplemetary Fig. 3. Results extracted from the simulated FRF of the T-shaped structure contaminated with a SNR of 60dB. (**a**, **b**) poly-reference Least Squares Complex Frequency (pLSCF) and (**c**, **d**) poly-reference Complex Frequency (pCF) stabilization plots constructed from the Frequency Response Function (FRF) contaminated with noise with a Signal-to-Noise Ratio (SNR) of 60dB by identifying models with order, n, ranging from 1 to 100. (**b**, **d**) details of the two closely spaced modes around 10.5 Hz. (**c**) Modal Assurance Criterion (MAC) between the Finite Element (FE) modal vectors and those estimated with the pLSCF technique. (**d**) MAC between the FE modal vectors and those estimated with the proposed pCF approach.

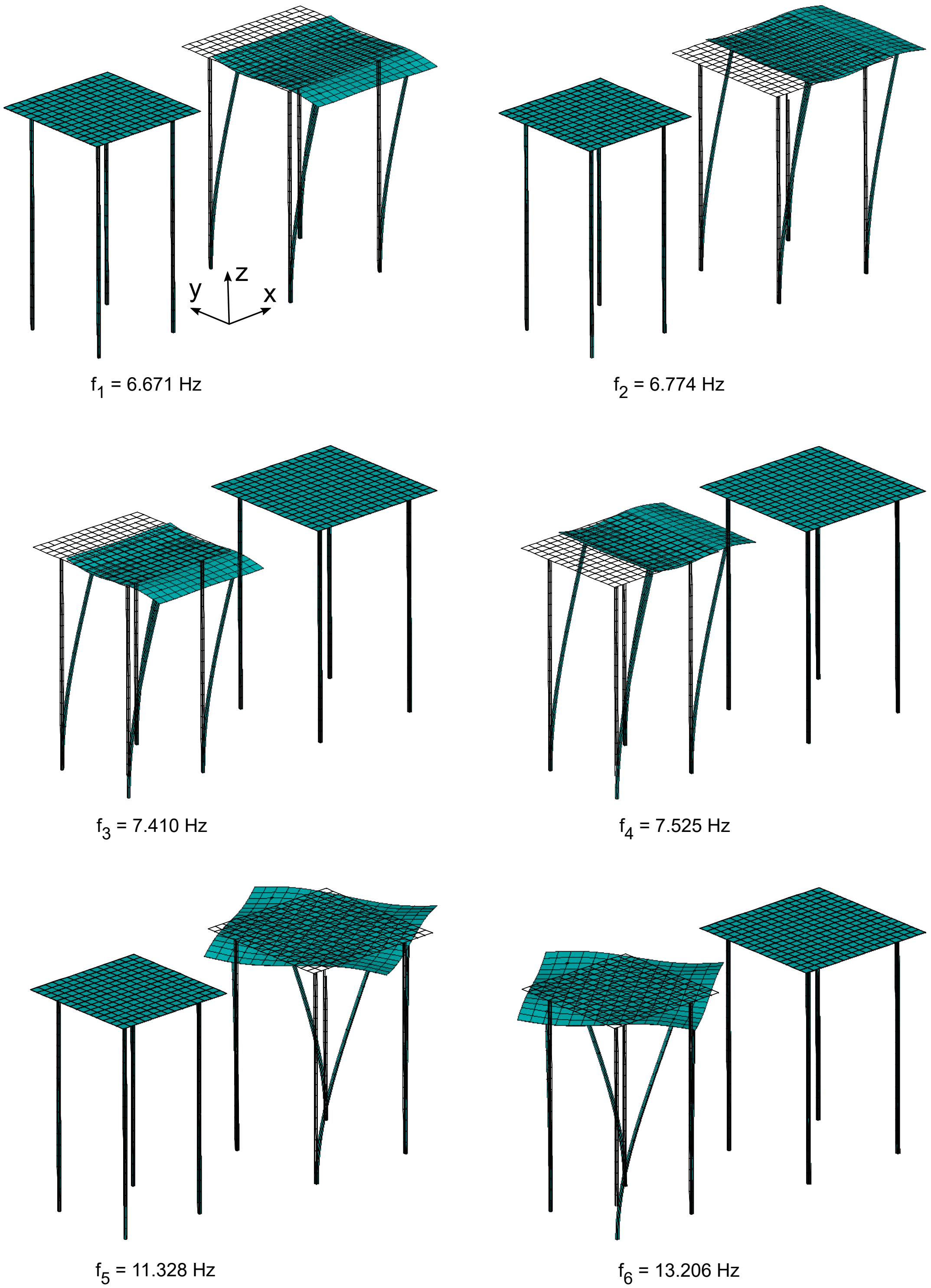

Supplemetary Fig. 4. Simulated modal properties of the two-platform specimen. Exact natural frequencies and mode shapes of the two platforms obtained with its Finite Element model.

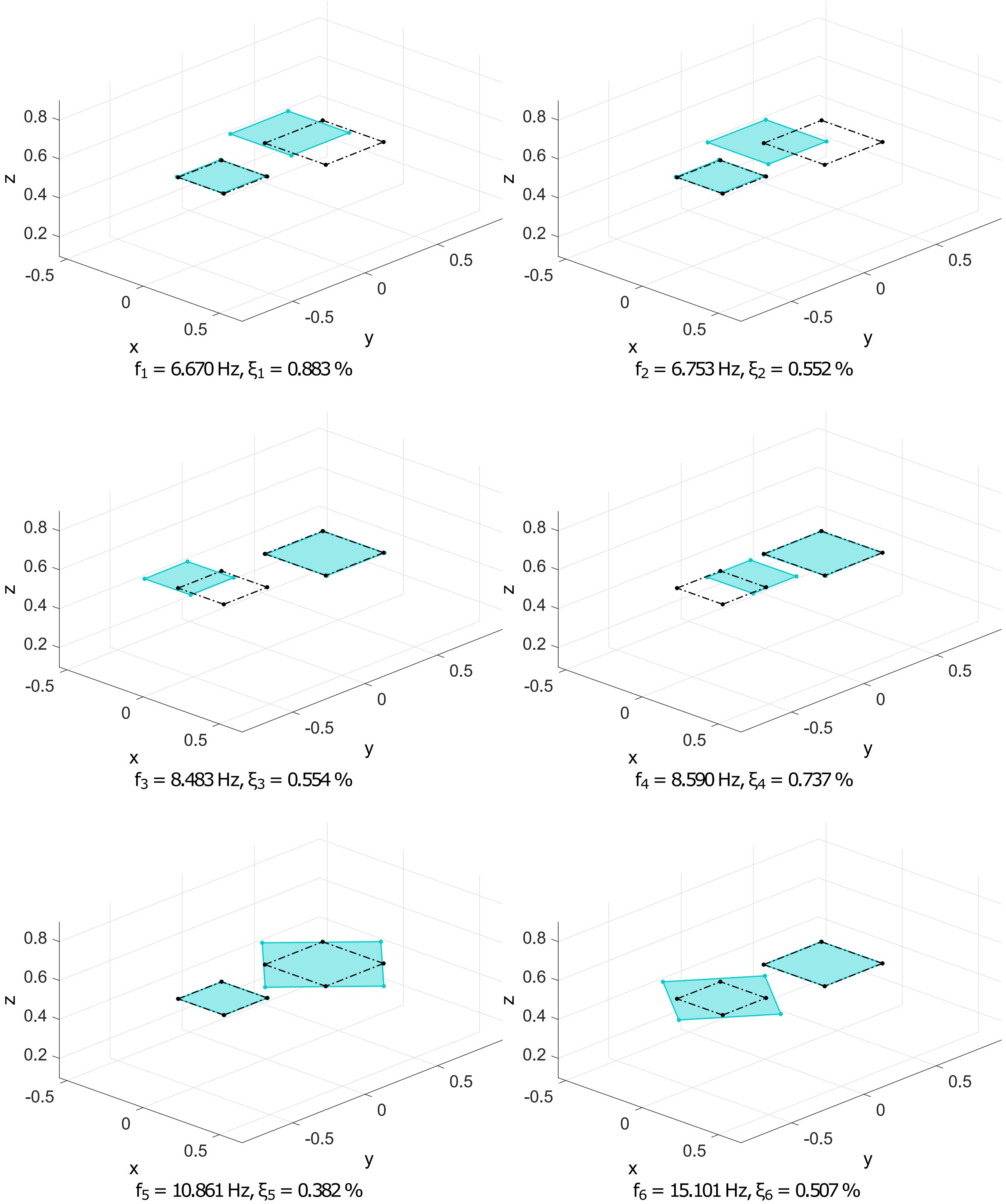

Supplemetary Fig. 5. Identification results obtained with the ITD method for the two-platform specimen. Natural frequencies, damping ratios and mode shapes of the two platforms identified with the Ibrahim Time Domain (ITD) method.

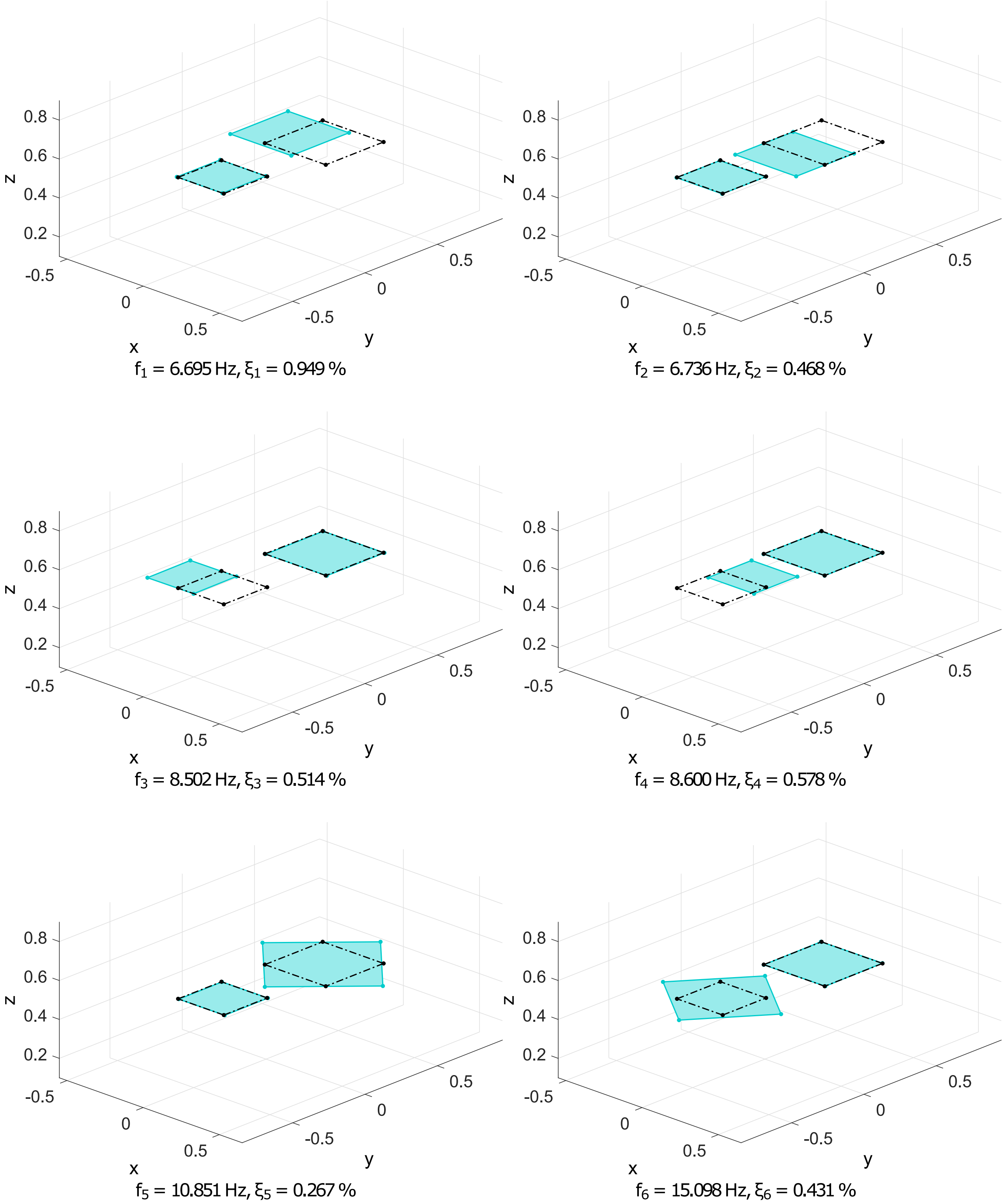

Supplemetary Fig. 6. Identification results obtained with the pLSCF method for the two-platform specimen. Natural frequencies, damping ratios and mode shapes of the two platforms identified with the poly-reference Least Squares Complex Frequency (pLSCF) method.

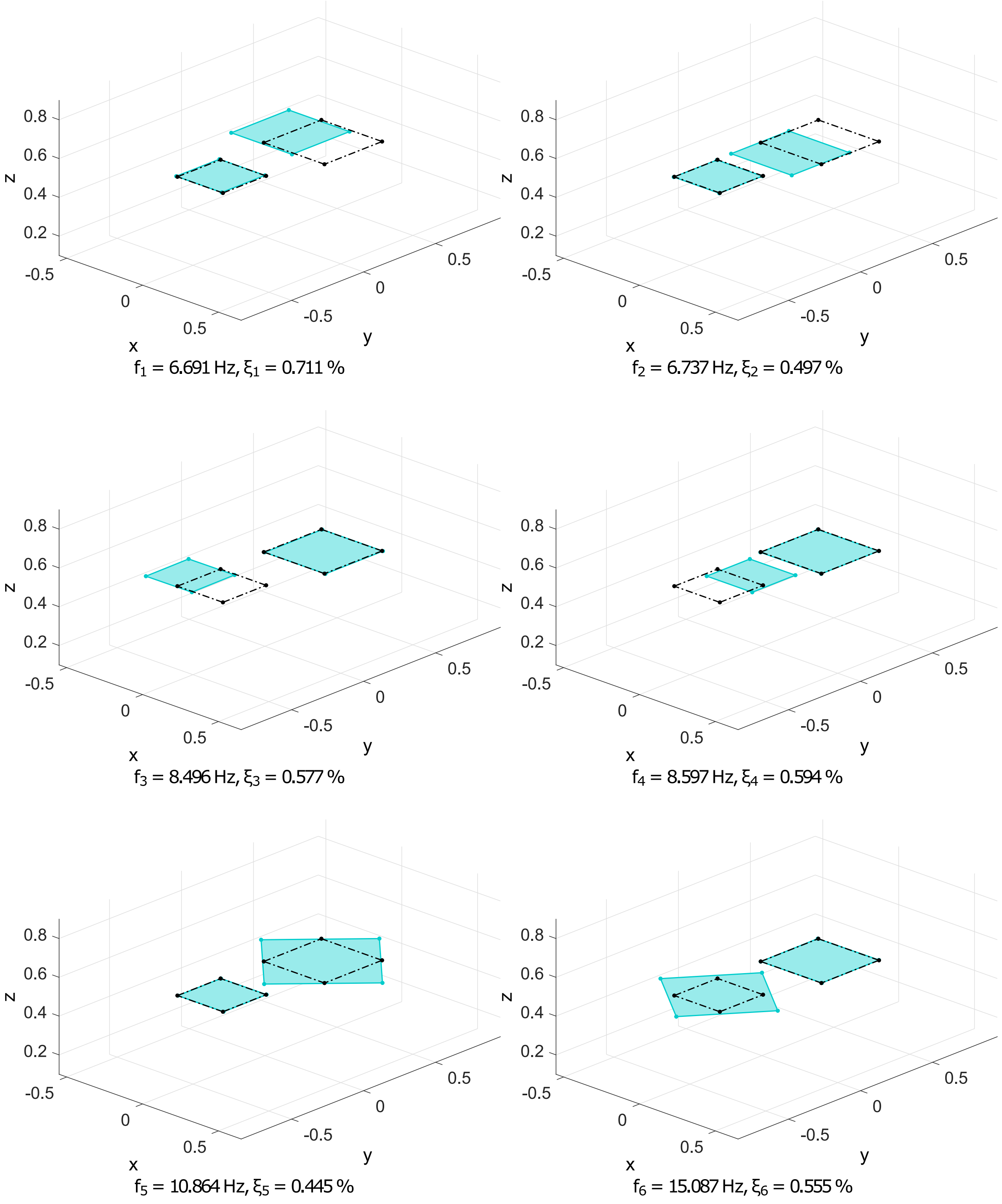

Supplemetary Fig. 7. Identification results obtained with the pCF method for the two-platform specimen. Natural frequencies, damping ratios and mode shapes of the two platforms identified with the proposed poly-reference Complex Frequency (pCF) method.

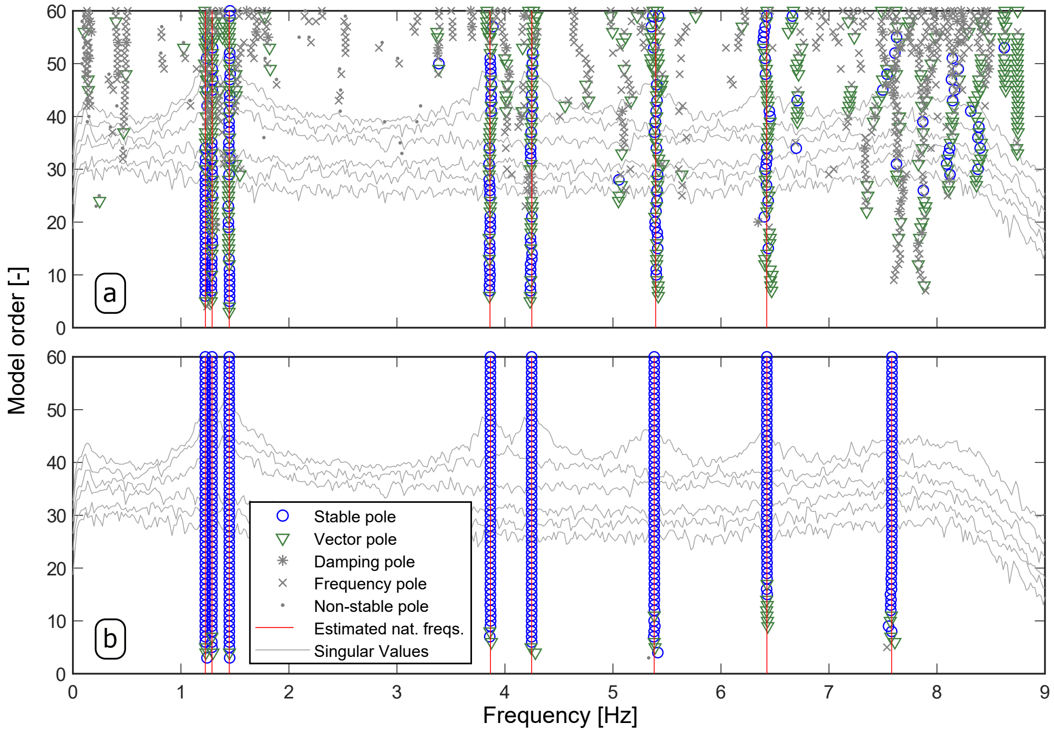

Supplemetary Fig. 8. Stabilization diagrams constructed from the vibration responses of the HCT stored in dataset 1. (**a**) poly-reference Least Squares Complex Frequency (pLSCF) and (**b**) poly-reference Complex Frequency (pCF) results obtained from the vibration responses of the Heritage Court Tower (HTC) recorded in dataset 1 by identifying models with order, n, ranging from 1 to 60.

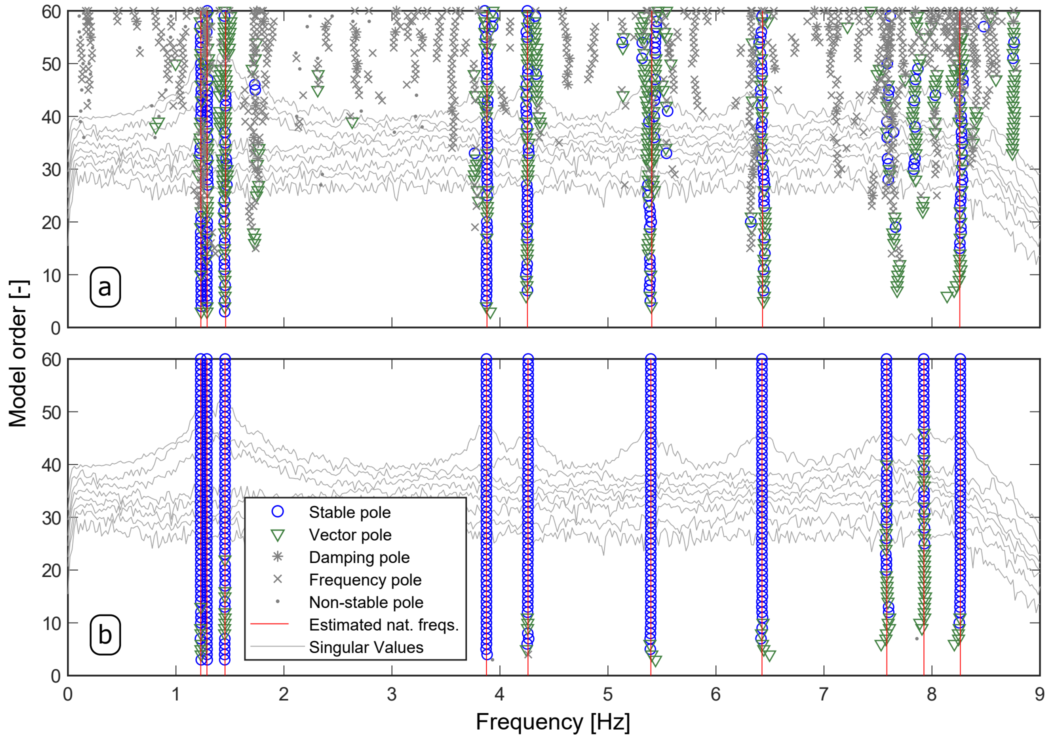

Supplemetary Fig. 9. Stabilization diagrams constructed from the vibration responses of the HCT stored in dataset 2. (**a**) poly-reference Least Squares Complex Frequency (pLSCF) and (**b**) poly-reference Complex Frequency (pCF) results obtained from the vibration responses of the Heritage Court Tower (HTC) recorded in dataset 2 by identifying models with order, *n*, ranging from 1 to 60.

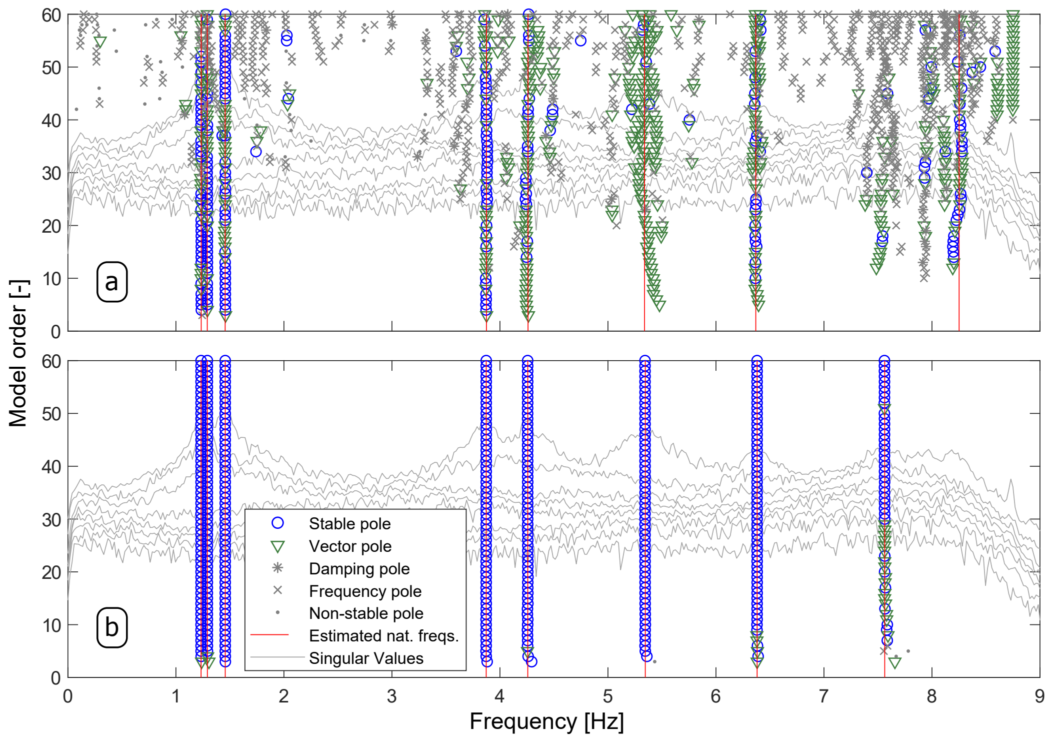

Supplemetary Fig. 10. Stabilization diagrams constructed from the vibration responses of the HCT stored in dataset 3. (**a**) poly-reference Least Squares Complex Frequency (pLSCF) and (**b**) poly-reference Complex Frequency (pCF) results obtained from the vibration responses of the Heritage Court Tower (HTC) recorded in dataset 3 by identifying models with order, *n*, ranging from 1 to 60.

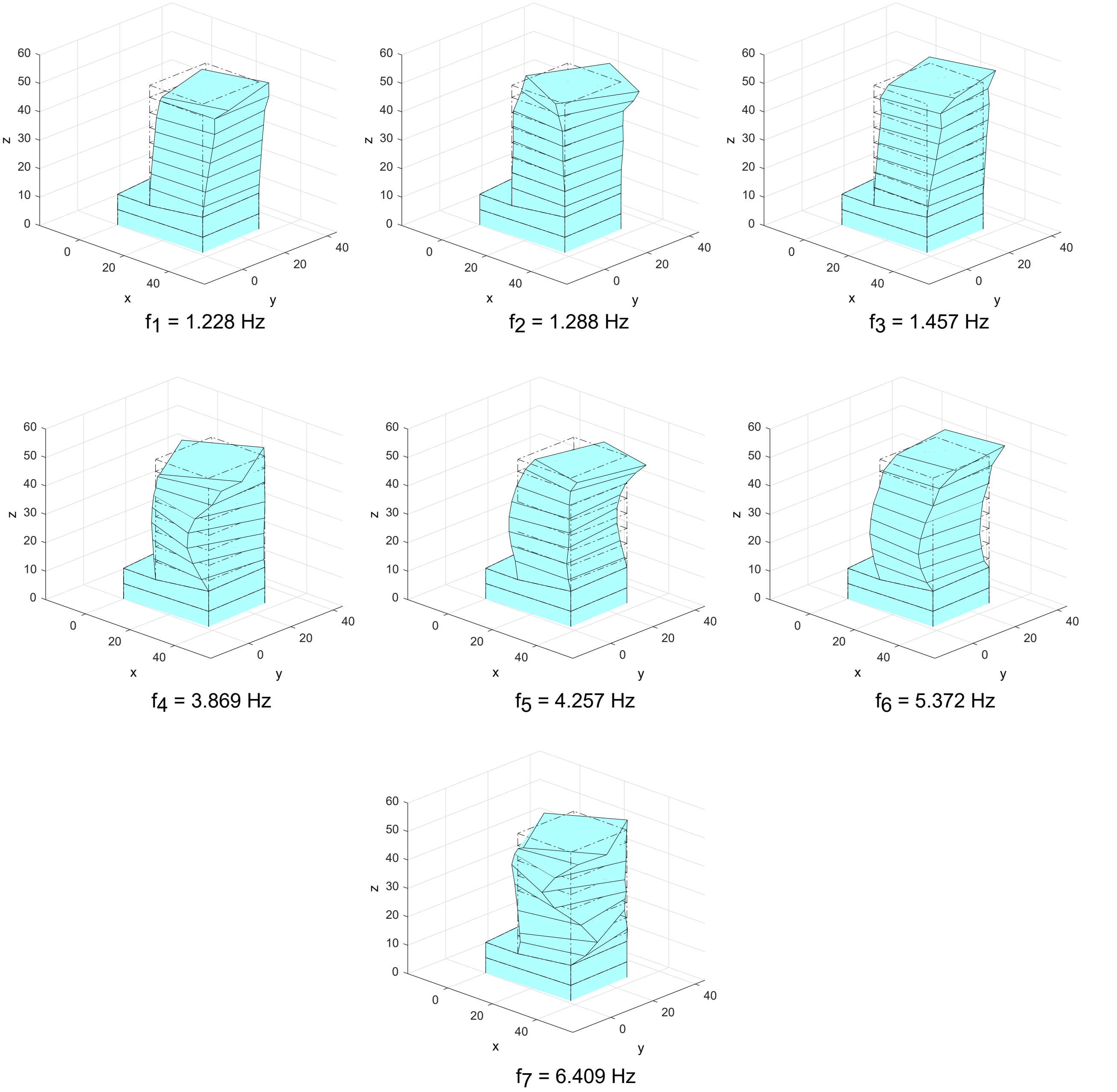

Supplemetary Fig. 11. Global identification results for the Heritage Court Tower obtained with the pLSCF method. Natural frequencies and damping ratios, and global mode shapes estimated with the poly-reference Least Squares Complex Frequency (pLSCF) identification method.

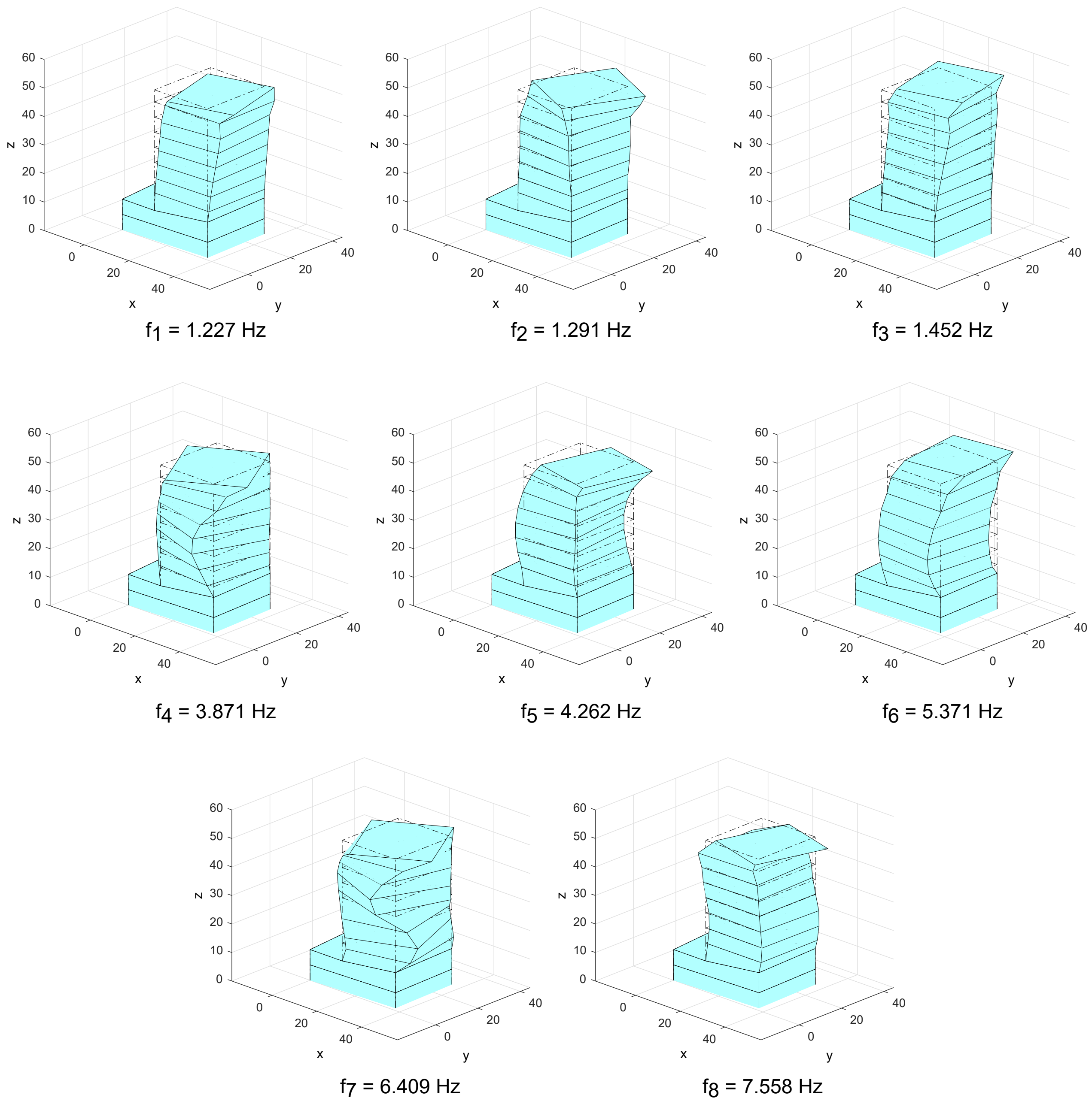

Supplemetary Fig. 12. Global identification results for the Heritage Court Tower obtained with the pCF method. Natural frequencies and damping ratios, and global mode shapes estimated with the proposed poly-reference Complex Frequency (pCF) identification method.

II. Supplementary Tables

Supplemetary Tab. 1. Exact mode shape vectors of the T–shaped Structure

DOF/Mode	1	2	3	4	5	6	7	8	9	10
S01	1.546E-01	-3.225E-10	6.510E-13	2.825E-01	-1.920E-11	-1.030E+00	1.848E-02	-8.471E-13	6.185E-12	-9.947E-01
S02	3.364E-10	1.562E-01	-1.518E-12	1.776E-11	7.801E-01	-9.216E-12	1.000E+00	5.555E-01	-7.210E-01	-5.669E-12
S03	5.338E-01	-1.106E-09	1.762E-11	4.731E-01	-2.453E-11	-6.634E-01	-4.952E-02	6.483E-13	-6.057E-12	1.000E+00
S04	1.161E-09	5.357E-01	-1.698E-11	2.972E-11	1.000E+00	-6.468E-12	3.239E-01	-3.071E-01	6.049E-01	5.082E-12
S05	2.175E-09	9.952E-01	-4.994E-11	-1.279E-12	-2.816E-01	1.790E-12	-2.550E-02	-3.841E-01	-6.847E-01	-4.558E-12
S06	1.940E-09	1.000E+00	1.000E+00	3.402E-11	-4.227E-01	3.222E-14	-5.600E-01	1.000E+00	1.000E+00	7.142E-12
S07	-4.862E-01	9.773E-10	-5.016E-11	1.000E+00	-4.069E-11	1.000E+00	-1.401E+12	2.680E-12	-4.282E-12	-4.244E-01
S08	1.000E+00	-2.056E-09	5.780E-11	-2.164E-02	8.016E-12	3.319E-01	1.615E-02	-1.625E-13	1.486E-12	-2.342E-01
S09	2.431E-09	1.000E+00	-1.000E+00	-3.702E-11	-4.227E-01	1.189E-11	-5.668E-01	1.000E+00	1.000E+00	4.897E-12
S10	4.862E-01	-9.781E-10	5.017E-11	-1.000E+00	4.428E-11	-1.000E+00	-1.401E+12	-1.872E-12	2.912E-12	4.244E-01
